# A public human population of TRAV26+ type II natural killer T cells recognises CD1d in a lipid-independent manner

**DOI:** 10.1038/s41467-026-78124-5

**Published:** 2026-10-07

**Authors:** Kean Chan Yew Poa, Christopher M. Harpur, Tan-Yun Cheng, Elena Batleska, Catriona V. Nguyen-Robertson, Rajesh Lamichhane, Alison A. White, Geovane Marques-Ferreira, Caroline Soliman, Scott JJ Reddiex, Christopher Menne, Adam P. Uldrich, D. Branch Moody, Jamie Rossjohn, Dale I. Godfrey, Daniel G. Pellicci, Jérôme Le Nours, Catarina F. Almeida

**Affiliations:** 1 Infection and Immunity Program and Department of Biochemistry and Molecular Biology, Biomedicine Discovery Institute, Monash University, Clayton, VIC Australia; 2 Department of Microbiology & Immunology, Peter Doherty Institute for Infection and Immunity, The University of Melbourne, Melbourne, VIC Australia; 3 Infection, Immunity and Global Health, Murdoch Children’s Research Institute, Parkville, VIC Australia; 4 Division of Rheumatology, Inflammation, and Immunity, Brigham and Women’s Hospital and Harvard Medical School, Boston, MA USA; 5 Departamento de Microbiologia, Instituto de Ciências Biológicas, Universidade Federal de Minas Gerais, Belo Horizonte, Brazil; 6 Department of Paediatrics, The University of Melbourne, Melbourne, VIC Australia; 7 Present Address: CSL Innovation Pty Ltd., Melbourne, VIC Australia

**Keywords:** NKT cells, Cellular immunity

## Abstract

Most studies of CD1d-restricted natural killer T cells (NKT) have focused on type I *TRAV10-TRAJ18*^+^ NKT cells that recognise the glycolipid α-GalCer. Our understanding of type II NKT cells, their T cell receptor (TCR) gene usage, and their ligand specificity and diversity remains unclear. Here, using CD1d tetramers carrying diverse endogenous lipids (CD1d-endo), we identified public human TRAV26^+^ type II NKT TCRs present in all examined individuals, with many expressing a conserved *TRAV26-2-TRAJ48* TCRα chain. Cellular, molecular and lipidomic analyses showed that TRAV26^+^ TCRs bound with similar affinities to CD1d loaded with diverse lipids, suggesting lipid-independent binding. Crystal structures of TRAV26^+^ TCR-CD1d complexes suggested these TCRs bound solely to the A’-roof of CD1d, distal to the protruding lipid. Collectively, we have uncovered a population of public lipid-independent TRAV26^+^ type II NKT cells.

## Abstract (web summary)

Most studies on lipid-reactive unconventional T cells have focused on type 1 NKT cells expressing a semi-invariant T cell receptor (TCR) recognising α-galactosylceramide. Here, using CD1d-tetramers carrying mammalian-endogenous self-lipids, the authors identify public human type II NKT cells, many expressing a conserved TCRα chain across different individuals.

## Introduction

Natural Killer T (NKT) cells recognise lipid-based antigens (Ag) presented by the monomorphic MHC class I-like molecule CD1d. NKT cells are generally classified into two main groups, type I and II NKT cells^1^. Most studies have focused on type I NKT cells, which express an evolutionarily conserved public T cell receptor (TCR) repertoire in humans, defined by an invariant *TRAV10-TRAJ18*^+^ TCRα chain paired with *TRBV25-1*^+^ TCRβ chains, that confers co-recognition of a prototypical glycolipid Ag known as α-galactosylceramide (α-GalCer) and related hexosyl ceramides when bound to CD1d^2,3^. The use of CD1d-α-GalCer tetramers has enabled extensive research to characterise the phenotypic profile and function of type I NKT cells^1,2^.

Type II NKT cells were identified circa 30 years ago in mice as CD4^+^ T cells that do not recognise MHC class II^4,5^, or as CD1d-reactive cells that do not require CD1d recycling to endosomes^6^. Those studies suggested that type II NKT cells express diverse TCR genes, distinct from the semi-invariant TCR characteristic of type I NKT cells, and are therefore commonly described as ‘diverse’ NKT cells^1^. In contrast to type I NKT cells, type II NKT cells do not recognise α-GalCer but can be activated by other CD1d-bound lipid-based Ags including sulfatides and phospholipids as well as small molecules^2,5,7^. Type II NKT cells appear to play a unique role in the immune system^2^, with some studies in humans suggesting opposing roles to type I NKT cells^8,9^. Type II NKT cells are comprised of specialised subsets capable of monitoring biological processes that impact on lipid metabolism and consequently, on the self-lipids bound to CD1d^5,10–13^. These cells are associated with, or implicated in, various diseases such as hepatitis, ulcerative colitis, and cancer^8,9^. In humans, type II NKT cells appear to outnumber type I NKT cells^14–16^. However, the paucity of reagents to clearly identify type II NKT cells has left fundamental questions unanswered that include the identity of immunodominant Ags, TCR patterns, the underlying mechanisms that regulate their numbers and the molecular mechanisms of Ag recognition^2^.

Insight has been gained into the molecular mechanism that underpins the recognition of CD1d-lipid complexes by human and mouse type I NKT TCRs^17,18^, as well as mouse type II NKT TCRs^19–21^ and ‘atypical’ NKT cells^22–26^. These studies led to the fundamental principle that underscored the NKT TCR co-recognition paradigm of CD1d-lipid complexes, whereby the NKT TCR contacts both CD1d and the presented Ag^3^. However, further work is required to establish the molecular basis of CD1d-lipid recognition by human type II NKT TCRs.

Here, using human CD1d tetramers presenting endogenous lipids, we identify a population of public type II NKT cells that express a TRAV26^+^ TCRα chain. Lipidomic scanning of CD1d-TRAV26^+^ TCR complexes revealed a diverse array of trapped lipids, and structural determination of the ternary complex showed that the TCR contacted CD1d in an Ag-independent manner.

## Results

### Identification of human CD1d-endo-reactive NKT cells

**Fig. 1:**
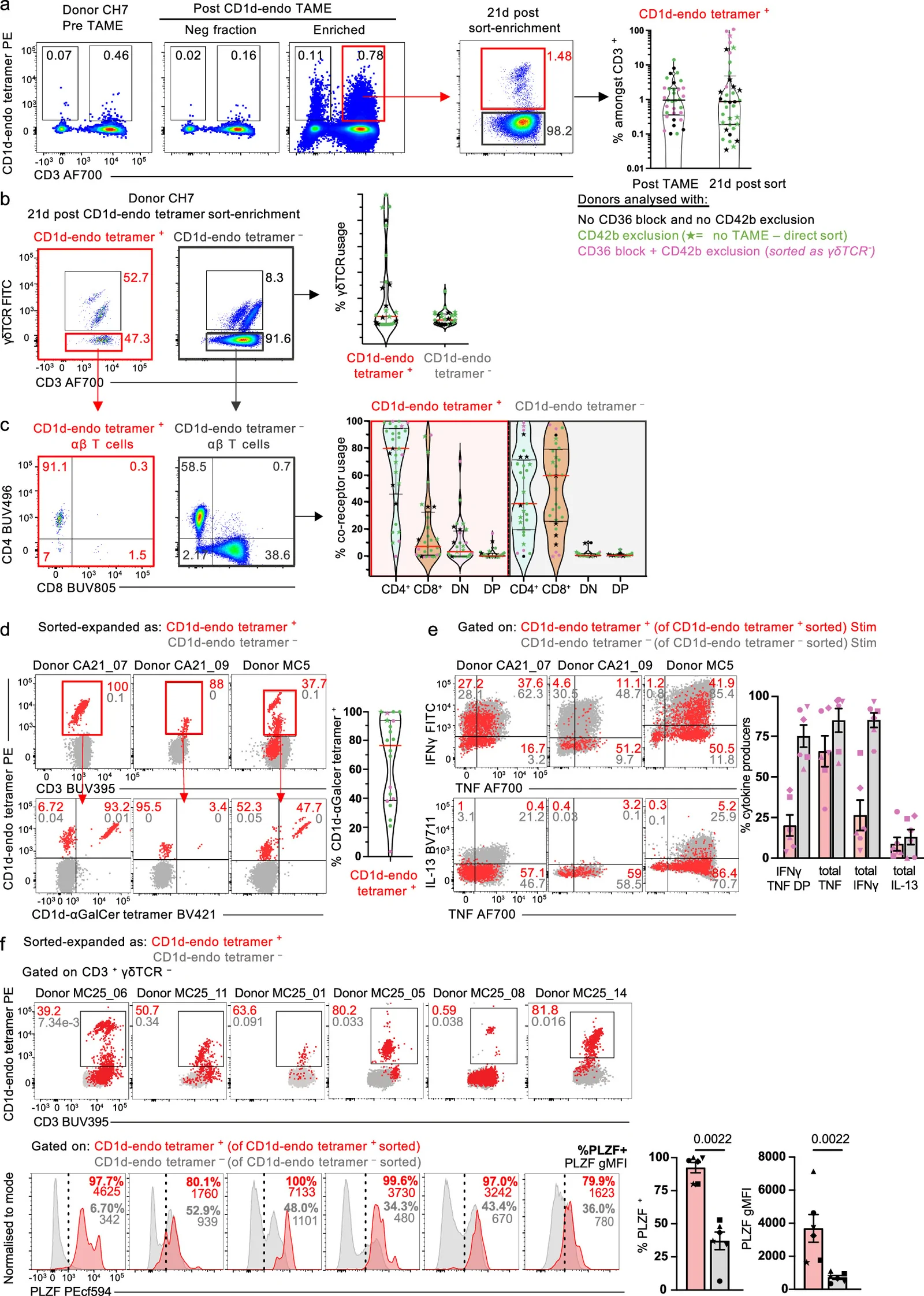
Identification of CD1d-endo-reactive T cells in PBMCs. **a** Representative flow cytometry plots showing pre- and post-CD1d-endo tetramer-associated magnetic enrichment (TAME), gated on 7AAD^−^CD14^−^CD19^−^CD42b^-^ single lymphocytes. CD1d-endo tetramer^+^CD3^+^ cells (red gate) were sorted and expanded in vitro for 21 days and reassessed for their ability to bind CD1d-endo tetramers. Violin plots depict median ± interquartile range (IQR) of CD1d-endo tetramer^+^ within CD3^+^ cells. Symbols represent individual donors (33 over 19 experiments post-TAME, 37 over 20 experiments post sort-expansion), coloured according to whether CD36 block was performed prior to CD1d-tetramer staining (pink) and/or CD42b^+^ cells excluded (green). Stars = no TAME. Post-expansion, both CD1d-endo tetramer^+^ (red) or CD1d-endo-tetramer^-^ (dark grey) populations were assessed for expression of γδ TCR (**b**, *n* = 27 donors over 15 experiments), and CD4 or CD8 within the γδ TCR^-^ αβ T cell compartment (**c**, *n* = 29 donors over 18 experiments), with data summarised in the violin plots, coloured as per (**a**). **d** Representative overlay dot plots showing CD3 versus CD1d-endo tetramer (top) or CD1d-endo tetramer versus CD1d-α-GalCer tetramer co-labelling (bottom) in expanded cells, sorted as CD1d-endo tetramer^+^ CD3^+^ (red) or sorted as CD1d-endo tetramer^-^ CD3^+^ (grey underlay). Violin plot shows median ± IQR of CD1d-α-GalCer tetramer^+^ co-labelling of CD1d-endo tetramer^+^ expanded cells (20 donors over 16 experiments). **e** Overlay dot plots show TNF *versus* IFNγ or IL-13 intracellular staining on stimulated CD1d-endo tetramer^+^ cells (red) or CD1d-endo tetramer^–^ (grey). Quadrant placement was based on unstimulated controls (21 days post-sort expansion). Bar graphs summarise cytokine production percentages ± 95% confidence intervals from 6 donors across 4 experiments. The symbols in (**b-e**) are coded as per (**a**). **f** Overlay dot plots showing CD3 versus CD1d-endo tetramer (top) on expanded cells (gated as γδ TCR^-^) that were sorted as CD1d-endo tetramer^+^ CD3^+^ (red) or CD1d-endo tetramer^-^ CD3^+^(grey) following CD36 blocking and CD42b exclusion. Numbers indicate percentages of cells within the gates. Overlay histograms below show intracellular PLZF expression by cells gated as γδ TCR^-^ and CD1d-endo tetramer^+^ (in red, from CD1d-endo tetramer^+^ sorted cells) or CD1d-endo tetramer ^–^ (in grey, from CD1d-endo tetramer^-^ sorted cells). Numbers indicate PLZF% and gMFI below. Dashed line represents gate placement for% determination. Bar graphs summarise PLZF% and gMFI ± SEM from *n* = *6* donors analysed across 3 experiments. ** p = 0.0022 for a two-tailed Mann-Whitney U test performed in both graphs (PLZF% or gMFI).

Early studies suggested that type II NKT cells can recognise and respond to CD1d carrying endogenous lipids (CD1d-endo)^4,6,14–16,27,28^. Although some target lipid-Ags for type II NKT cells are known, the study of these cells has been hindered by a limited understanding of their target lipid repertoire. To further investigate the antigenic targets and diversity of human type II NKT cells, CD1d-endo tetramers were generated using glycosylation-deficient GnTI^−/−^ Human Embryonic Kidney 293 (HEK293S) cells to express soluble CD1d ectodomains that incorporate a cellular cargo of endogenous lipids during synthesis^29,30^. These CD1d-endo tetramers stained over 0.1% of live CD3^+^ T cells within peripheral blood mononuclear cells (PBMCs) from healthy human donors, albeit with varied and often low mean fluorescence intensity (MFI) in many cases (Fig. 1a and S1a). After one round of tetramer-associated magnetic enrichment (TAME)^31^ using phycoerythrin (PE)-labelled CD1d-endo tetramers, the frequency of the CD1d-endo tetramer^+^ amongst CD3^+^ T cells generally yielded more defined populations at proportions ranging up to 13% (Median (M) 0.92%, interquartile range (IQR) 0.3-1.9%), in contrast to PE-conjugated streptavidin (SAV-PE) negative controls where no clear population was identified (Fig. S1a). However, it was possible that some of these frequencies were impacted by non-TCR CD1d ligands such as CD36 family members^32^, as suggested by the enrichment of CD3^-^ cells capturing the CD1d-tetramer after TAME (Fig. 1a and S1a-b). To minimise this, for many samples, we used a CD42b exclusion gate to exclude platelets which express high levels of CD36 and/or CD36 blocking antibody prior to staining of some samples.

To confirm TCR-dependent CD1d-binding specificity, CD3^+^ CD1d-endo tetramer^+^ cells were enriched using flow cytometric cell sorting and expanded in culture for 18-20 days and re-stained with CD1d-endo tetramers. The CD1d-endo tetramer^+^ populations varied widely in frequency (0.035-100%) of CD3^+^ cells (Fig. 1a) but often formed more distinct clusters (M = 0.84%, IQR = 0.2-4.5%), when compared to post-TAME samples. This may be because the expanded cells were devoid of platelets or other cell types that, through cell adhesion and/or trogocytosis, may confer TCR-independent binding with CD1d-tetramers (Fig. 1 and S1a)^32^. CD1d-endo tetramer^+^ cells included both αβ and γδ T cells, with γδ T cell frequencies varying widely in the 27 donors analysed. CD1d-endo-tetramer^+^ αβ T cells in these cultures (Fig. 1b) were skewed towards CD4^+^ single positive (SP) expression (M 80%, IQR 46-94%), although CD8^+^ SP and CD4^-^CD8^-^ double negative (DN) populations were also detected at lower frequencies (Fig. 1c). Conversely, the CD1d-endo tetramer^–^ conventional T cells had similar frequencies of CD4^+^ SP and CD8^+^ SP T cells.

By co-labelling expanded cells with CD1d-endo tetramer and CD1d-α-GalCer tetramer, many CD1d-binding cells (M 76.5%, IQR 38-94%) co-stained with both tetramers (Fig. 1d). In the three donors shown, representative of 20 analysed this way, the staining pattern revealed distinct T cell clusters that likely reflected the oligoclonality of expanded NKT cells. Most, but not all, clusters showed a positive correlation for staining intensity by both tetramers, suggesting that binding to CD1d occurs regardless of the lipids within the CD1d cleft.

The functional potential of expanded cells was also investigated. After stimulation with phorbol 12-myristate 13-acetate (PMA) and ionomycin, CD1d-endo tetramer^+^ cells expressed tumour necrosis factor (TNF) and interferon-γ (IFNγ) (mean 66% and 26%, respectively (Fig. 1e), and cytokine production was detected in both CD8^-^ and CD8^+^ cells (Fig. S1c). While some CD1d-endo tetramer^+^ cells co-produced these cytokines (mean 20%), some produced either one or the other (Fig. 1e). IL-13 was only detected in a small subset of CD1d-endo tetramer^+^ cells in some donors, in contrast to CD1d-endo tetramer^-^ cells where IL-13 was more frequently detected (Fig. 1e). Thus, type II NKT cell populations capable of producing pro-inflammatory cytokines are readily identified among human donors by CD1d-endo tetramers with no defined exogenously loaded antigen.

Next, we asked whether CD1d-endo tetramer sorted and expanded cells expressed promyelocytic leukaemia zinc finger (PLZF), encoded by the Zbtb16 gene, which is considered a master transcription factor associated with type I NKT cells and other innate-like T cell lineages^1,2^. In all 6 donors screened, PLZF was consistently expressed by CD1d-endo-tetramer^+^ cells (80-100%), with geometric mean fluorescence intensities that were 2-10-fold higher than those of CD1d-endo-tetramer^–^ cells, that were sorted and expanded in parallel (Fig. 1f). Notably, the latter also contained some PLZF^+^ populations (ranging 7-53%), perhaps reflecting the expansion of an unconventional T cell population, or a memory T cell subset. Overall, our results suggest that CD1d-endo reactive cells, including those co-binding CD1d-α-GalCer tetramers, share functional and developmental similarities to type I NKT cells, and other innate-like T cell lineages such as MAIT cells and γδT cells^1,2^.

### TRAV26 TCR bias amongst human CD1d-endo-reactive T cells

**Fig. 2:**
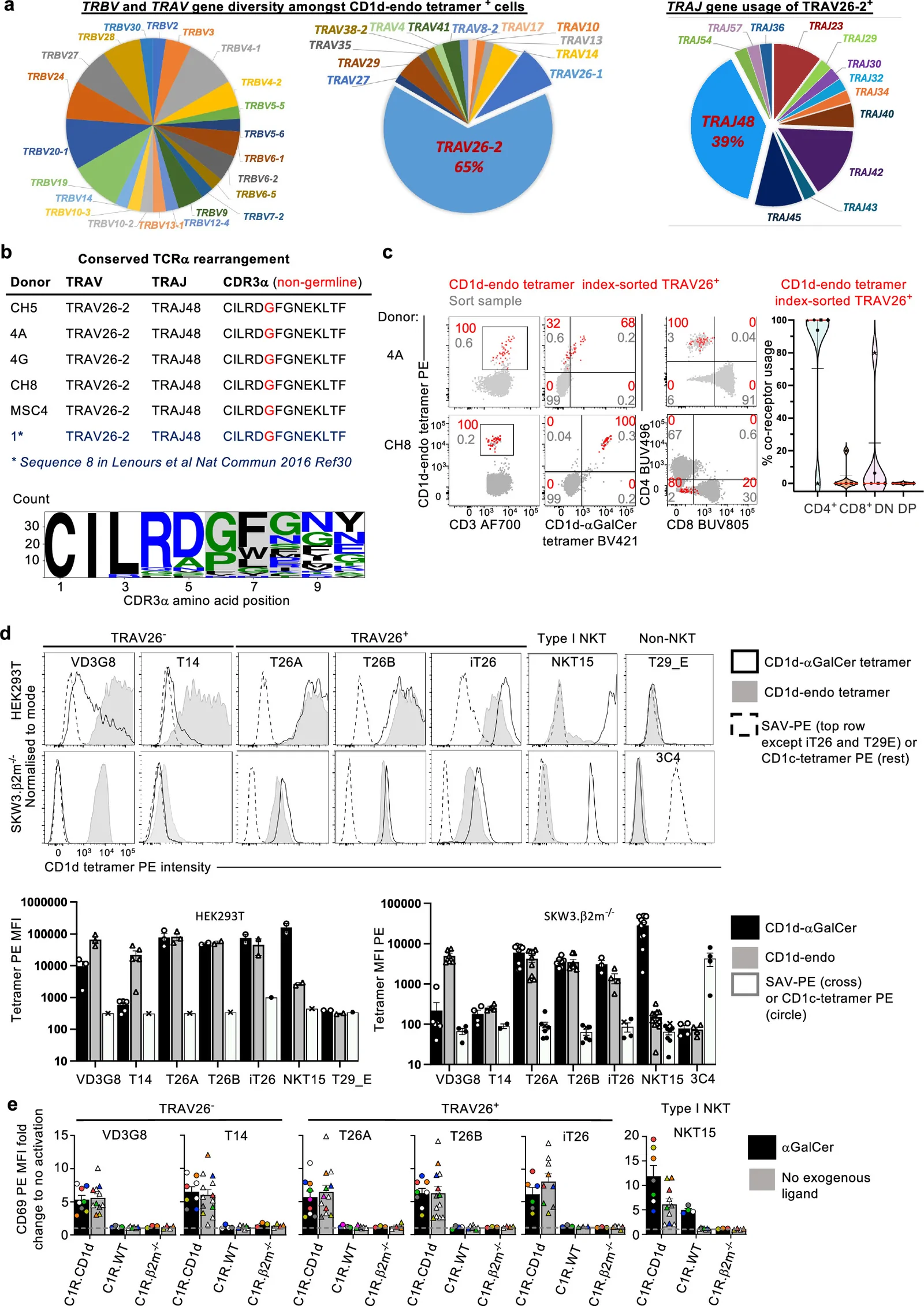
TCR usage amongst CD1d-endo tetramer^+^ T cells. CD1d-endo tetramer^+^ T cells were single-cell sorted by flow cytometry and TCR sequences determined by nested PCR. **a** Pie charts showing *TRAV and TRBV* gene segment diversity, and *TRAJ* gene usage amongst TRAV26-2^+^ cells derived from 14 donors over 7 experiments. **b** Invariant *TRAV26-2-TRAJ48* rearrangement identification across different donors, and sequence conservation of the CDR3α from *n* = 39 TRAV26-2^+^ clones. Hydrophobic (green) and charged (blue) residues are shown alongside the most common positions for non-germline encoded residues (grey highlight). **c** Representative overlay dot-plots of index-sorted TRAV26^+^ (red) and total T cells post-culture (grey). Violin plot summarises the CD4/CD8% distribution with median ± interquartile-range of TRAV26^+^ cells index-sorted from 6 donors over 5 experiments. Five distinct TCR sequences (from Tables 1 and 2) were used to generate transiently transfected HEK293T cells (top) or TCR-transduced SKW3.β2 m^−/−^ clones (bottom). **d** Representative dot plots showing CD1d-endo and CD1d-α-GalCer tetramer staining of TRAV26^+^, and TRAV26^-^ clones. Type I NKT TCR^+^ clone NKT15 and non CD1d-reactive TCRs (T29_E-top/3C4-bottom) were included as controls. Bar graphs summarise CD1d-tetramer mean fluorescence intensity (MFI) ± SEM for: HEK293T-transfections (left), from *n* = 3 (VD3G8), *n* = 5 (T14), *n* = 3 (T26A) and *n* = 2 (T26B/iT26/NKT15/T29_E) independent experiments; or SKW3.β2 m^−/−^ (right), from *n* = 6 (VD3G8), *n* = 4 (T14/iT26, with *n* = 3 for CD1d-α−GalCer), *n* = 9 (T26A, *n* = 8 for CD1d-α-GalCer), *n* = 8 (T26B, with *n* = 7 for CD1d-α-GalCer), *n =* 11 (NKT15, *n =* 10 for CD1d-α-GalCer) and *n* = 3 (3C4). For *n* = 1 HEK293T experiments, staining controls included unconjugated SAV-PE (crosses) or CD1c tetramer-PE (spheres). For SKW3.β2m^−/−^ experiments, CD1c tetramer-PE controls were included in *n* = 4 (VD3G8) *n* = 2 (T14) *n* = 6 (T26A) *n* = 5 (T26B) *n* = 3 (iT26) *n* = 6 (NKT15) *n* = 4 (3C4) experiments, or SAV-PE for *n* = 1 (T26A/T26B/iT26/NKT15). **e** TCR-transduced cell lines were co-cultured with wild-type C1R cells (C1R.WT) or CD1d transduced (C1R.CD1d), as well as C1R.β2m^−/−^ cells in the presence or absence of α-GalCer (1μg/mL). Bar graphs show CD69 MFI fold increase ± SEM relative to SKW3.β2m^−/−^ alone. Each dot represents an independent experiment (with:without α−GalCer, respectively): *n* = 8:11 (VD3G8), *n* = 8:13 (T14), *n* = 9:12 (T26A), *n* = 8:13 (T26B), *n* = 6:9 (iT26), *n* = 7:10 (NKT15) in C1R.CD1d co-cultures; or *n* = 4:6 (VD3G8), *n* = 5:8 (T26A), *n* = 3:3 (iT26), *n* = 4:7 (T26B/T14/NKT15) in C1R.WT co-cultures; or *n* = 3:6 (iT26), *n* = 3:4 (T26A/T26B/T14/NKT15) in C1R.β2m^−/−^ co-cultures. Experiments shown in blue/grey/green/pink are from the series of experiments shown in Fig. S3a and those in orange/red/yellow are from Fig. S3b (isotype-control treated C1R.CD1ds, performed alongside C1R.β2m).

To characterise the TCR repertoire of the populations labelled by CD1d-endo tetramers, single CD1d-endo tetramer^+^ cells were index sorted from in vitro expanded cultures and their TCR sequences were determined^31^. Paired TCRα and TCRβ, or TCRδ and TCRγ chain-transcripts were determined for cells sorted as αβ T cells or γδ T cells (Tables 1 and 2). None of the sorted T cell clones contained the canonical TCR usage that define type I NKT TCRs. Instead, they exhibited a varied, yet biased, range of γδ and αβ TCR chains (Fig. 2a-b and Tables 1 and 2). Almost all γδ TCRs analysed incorporated *TRDV1* genes (17 of 18 Vδ sequences derived from 11 donors (Table 1), in line with previous studies that described Vδ1^+^ γδ T cells recognising CD1d^22,23,33^ and CD1c^34^. Unexpectedly, within the αβ TCRs, there was a strong bias towards TRAV26^+^ TCRs, with ~65% of αβ TCRs within CD1d-endo tetramer^+^ cells from 11 donors utilising *TRAV26-*2 gene segments. In addition, cells from 5 donors also utilised *TRAV26-1*, which differs from *TRAV26-2* by four germline encoded amino acids, as well as one amino acid in the CDR3α loop (Fig. 2a and Table 2). Further, *TRAV26-2 gene* segments were often associated with *TRAJ48* in 15 of 39 TCRs (Fig. 2a and Table 2), although other TRAJ gene-rearrangements were also detected, including *TRAJ42* in 6 of 39 TCRs, *TRAJ23* in 4 of 39 TCRs and *TRAJ45* in 4 of 39 TCRs (Fig. 2a and Table 2). Type II NKT cells possessing *TRAJ48* genes almost exclusively rearranged with *TRAV26*^+^, with 15 encoded by *TRAV26-2, one by TRAV26-1* and one by *TRAV29*. Moreover, a conserved CDR3α involving the insertion of the same amino acid (G in position 6) in the N-region during rearrangements between *TRAV26-2* and *TRAJ48* genes was identified in 5 donors across 5 independent experiments (Fig. 2b and Table 2), demonstrating public conservation of the CDR3α sequence. Notably, this same *TRAV26-2–TRAJ48* rearrangement had also been reported in a single NKT cell clone isolated via a different approach^25^. Another conserved motif was detected in positions 6 and 7 of the TRAV26-2 CDR3α sequences, where proline was also often seen at position 6, typically accompanied by a bulky residue at position 7 (Fig. 2b and Table 2). In contrast to the conserved TCRα chain, the TCRβ gene usage was highly diverse, as were the CDR3β sequences and lengths (Table 2).

**Table 1:** Paired γδ TCR sequences of CD1d-endo tetramer+ single-sorted cells

IMGT TCR gene nomenclatures and associated complementary determining region (CDR) loop amino acid sequences are shown for γδ T cells sorted with CD1d-endo tetramers post-sort/expansion in culture for 21 d as per Fig. 1a. The number of observations (Obs. column) as well as intra-donor frequency at which each unique clonotype was observed amongst total amplified γδ TCRs are shown from an analysis of 11 donors. A total of three sorting experiments were performed. Red residues are either partially or fully non-germline encoded. XX means undetermined. Sequences selected to generate γδ TCR-transduced NKT cell clones, are given a clone name. Amino acid positions: CDR1-IMGT (27 to 38), CDR2-IMGT (56 to 65), and CDR3 (105 to 117).

**Table 2:** Paired αβ TCR sequences of CD1d-endo tetramer+ single-sorted cells

IMGT TCR gene nomenclatures and associated complementary determining region (CDR) loop amino acid sequences are shown for αβ T cells sorted with CD1d-endo tetramers post-sort/expansion in culture for 21 d as per Fig. 1a. The number of observations (Obs. column) as well as intra-donor frequency at which each unique clonotype was observed amongst total amplified αβ TCRs are shown from an analysis of 14 donors. Sequences in bold contain the invariant TRAV26-TRAJ48 rearrangement. A total of eight single-cell sorting experiments were performed. Red residues are either partially or fully non-germline encoded. Sequences selected to generate αβ NKT cell clones are given a clone name. Amino acid positions: CDR1-IMGT (27 to 38), CDR2-IMGT (56 to 65), and CDR3 (105 to 117).

Single-cell index-sorting analysis of TCR gene expression revealed that the subpopulations co-staining with both CD1d-endo and CD1d-α-GalCer tetramers mainly comprised TRAV26^+^ TCRs, indicated by red dots in the two representative donors (Fig. 2c). These cells were also CD4^+^ (*M* = 100%) in 5 out of 6 donors analysed over 5 different index-sorting experiments, while in one donor, they were CD4^-^CD8^-^DN or CD4^-^CD8^low^ (Fig. 2c). In summary, the human CD1d-endo-reactive population harbours a public type II NKT repertoire characterised by biased *TRAV26*^*+*^ TCR usage and a semi-conserved CDR3α motif, often expressed as a canonical *TRAV26-2-TRAJ48* rearrangement.

### TRAV26^+^ TCRs directly recognise CD1d

To test the specificity of the CD1d-endo-reactive TCRs, five paired TCRs that included three TRAV26^+^ clones (‘T26A’, *TRAV26-2-TRAJ40 TRBV19*, ‘T26B’ *TRAV26-2-TRAJ42 TRBV13-1* and ‘iT26’, *TRAV26-2-TRAJ48 TRBV6-2*), and two TRAV26^–^ clones (an αβ TCR identified in ref. ^7^, ‘T14’, *TRAV14-TRAJ34 TRBV19*, and a γδ TCR ‘VD3G8’, *TRDV3-TRDJ2 TRG8*) (Tables 1 and 2) were transiently transfected into HEK293T cells, or transduced β2m-deficient SKW3 cells (SKW3.β2m^−/−^). These two TCR transduced cell systems were assessed for binding CD1d-α-GalCer and CD1d-endo tetramers, in comparison to a control cell line expressing a type I NKT TCR ‘NKT15’^35^, or non-NKT controls including the T29_E^7^ and the CD1c-reactive clone 3C4^32^ (Fig. 2d). The five lines stained strongly with CD1d-endo tetramers, with MFIs >45,000 in HEK293T or >1000 in SKW3.β2m^−/−^ cells (Fig. 2d), unlike the negative controls. While CD1d-α-GalCer tetramers showed much lower staining of the TRAV26^–^ lines (typically 10-100-fold less than CD1d-endo), they stained all three TRAV26^+^ lines to a similar degree as CD1d-endo tetramers (Fig. 2d), reflecting the co-staining profile observed after in vitro expansion of the parental clones (Fig. 2c). This suggested that the TRAV26^–^ TCRs may detect an undefined mammalian endogenous lipid presented by CD1d, and that α-GalCer can displace this lipid thereby preventing recognition, whereas the TRAV26^+^ lines are indifferent to the presence of α-GalCer. As expected, the type I NKT15 TCR line stained far more strongly with CD1d-α-GalCer tetramers (Fig. 2d), whereas the CD1d-restricted lines were not stained by SAV-PE alone, nor by CD1c-tetramers conjugated to PE (Fig. 2d), which instead specifically stained the 3C4 control.

To measure TCR-mediated activation following CD1d binding, NKT TCR-transduced cell lines were co-cultured with Ag-presenting cells with differing levels of CD1d. These included: wildtype C1R cells (C1R.WT) with low CD1d expression, CD1d transductants (C1R.CD1d) with high CD1d expression, or β2m knockout C1R cells (C1R.β2m^−/−^) that lack surface CD1d^7^. The TCR-transduced lines derived from CD1d-endo sorted cells upregulated the activation marker CD69 when co-cultured with C1R.CD1d, whereas C1R.WT or C1R C1R.β2m^−/−^ cells did not cause activation (Fig. 2e). α-GalCer elicited strong responses by the NKT15 cell line using C1R.WT or C1R.CD1d, whereas similar activation was observed in the presence or absence of α-GalCer for TRAV26^–^ or TRAV26^+^ lines (Fig. 2e). Notably, α-GalCer did not block activation of the TRAV26^–^ lines despite blocking CD1d tetramer staining of these lines (Fig. 2d), suggesting that α-GalCer was unable to saturate all CD1d molecules on the C1R Ag-presenting cells in these co-culture conditions. Thus, the identified TCRs confer signalling in response to CD1d, regardless of whether α-GalCer is present. Taken together, these data suggest that TRAV26^+^ NKT cells bind CD1d in a lipid-independent manner and represent a distinct population of human type II NKT cells.

We next assessed whether circulating CD14^+^ monocytes, which constitutively express higher levels of CD1d, compared to T cells cells^36^, could elicit activation of CD1d-endo reactive cells. To do this, we co-cultured a TRAV26 + NKT reporter line (T26A) with primary CD14^+^ monocytes (magnetically enriched using anti-CD14 beads), or donor matched CD11c^+^ monocyte-derived dendritic cells (MoDCs) and compared to co-cultures with C1R.CD1d, including a type I NKT line (NKT15) and α-GalCer or vehicle controls.

We detected clear CD1d surface expression (2.1-4.4 fold increase in CD1d gMFI) in MoDCs in 5 out of 10 donors, when compared to their pre-differentiation counterparts (CD14^+^ Mono, Fig. S4a, suggesting that a subset of CD1d^+^ MoDCs was being expanded, or CD1d upregulated, possibly reflecting different donor inflammatory status. Notably, CD1d levels were still lower than those CD1d-transduced C1Rs (8.3 to 69× lower gMFI).

In the absence of exogenous antigen, primary CD14^+^ cells were unable to activate T26A cells when co-cultured at a 1:1 ratio, whereas MoDCs elicited a slight but consistent increase in CD69 gMFI by both T26A and NKT15, 1.1–2.4-fold, and 1.2–2.7 fold-increase, compared to no activation, respectively, reflecting 35% and 41% CD69^+^ cells for each NKT line (Fig. S4b). However, this was inferior to the activation elicited by C1R.CD1d co-cultures co-cultured at similar 1:1 ratios, which led to 8.3-fold increases in CD69 gMFI mean for T26A (91% CD69^+^) or 12.3 fold for NKT15 (94% CD69^+^), or even when co-cultured at 1:10 C1R.CD1d:effector ratio (2.96-fold increase in CD69 gMFI/64% CD69^+^ for T26A, or 3.4 fold/71% CD69^+^ for NKT15). Panel C in Fig. S4 shows that indeed TCR-mediated activation of T26A (CD69 gMFI) significantly correlated to the levels of CD1d-surface expression on Ag-presenting cells, in the absence of exogenously added ligands.

In the presence of α-GalCer, both primary CD14^+^ cells as well as in vitro differentiated MoDCs elicited activation of NKT15 (2.5-4.8 fold-increases in CD69 gMFI/72% CD69^+^, and 2.5-11.1 fold/80% CD69^+^, respectively), demonstrating that their CD1d surface levels are sufficient to allow α-GalCer presentation and TCR-dependent activation of type I NKT cells (Fig. S4b).

Altogether, our results suggest that CD1d-expression in primary circulating monocytes alone is likely insufficient to elicit TCR-dependent activation of CD1d-endo reactive cells on its own, although certain inflammatory settings driving higher CD1d-surface expression may be able to overcome this.

### Trapping lipids between CD1d and TCRs

**Fig. 3:**
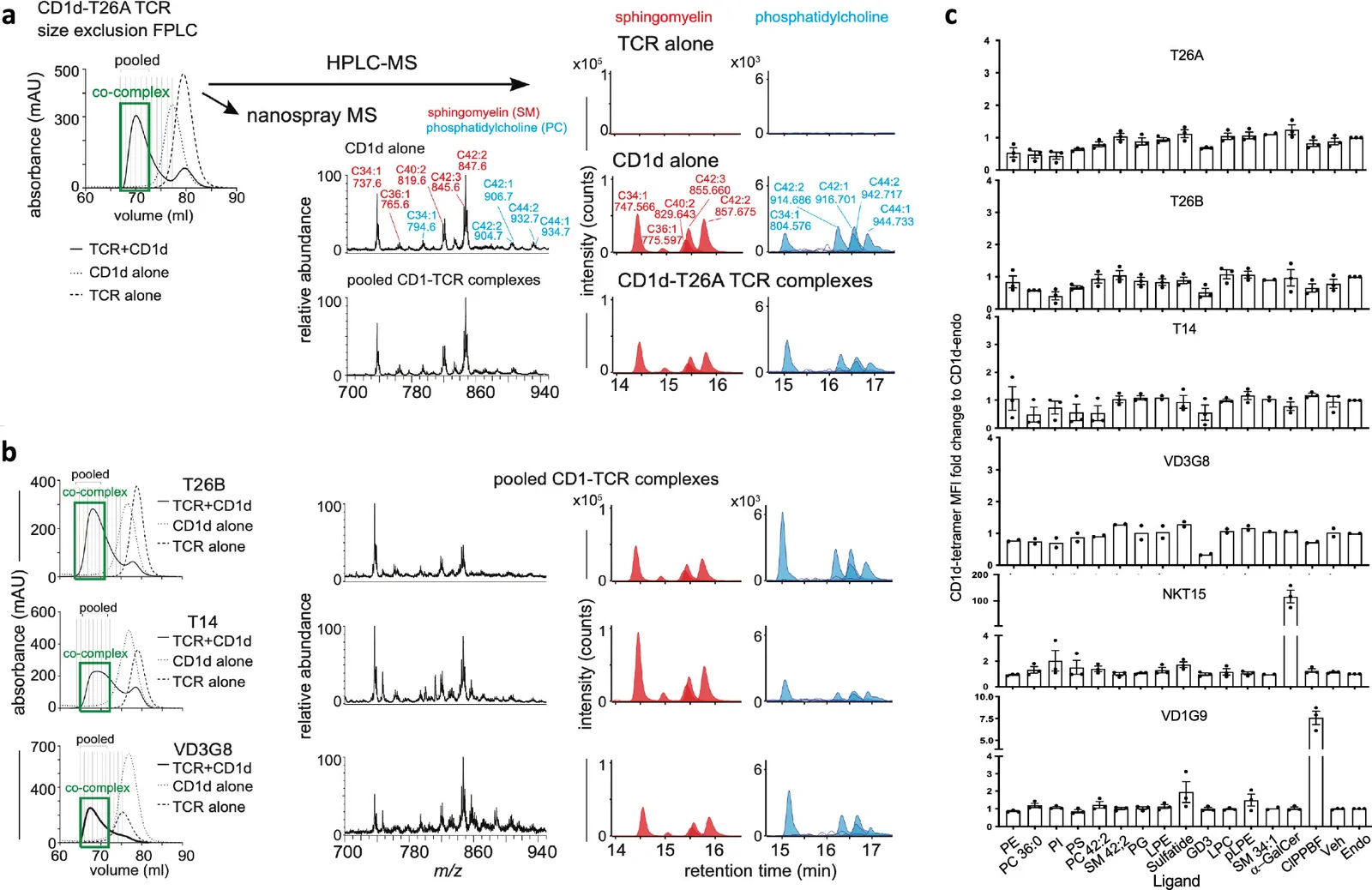
TCR trap determination of T26A TCR-CD1d-endo complexes. **(a)** CD1d alone, T26A TCR alone or CD1d-TCR complexes were introduced to size exclusion chromatography. For CD1d and TCR runs, fractions eluting earlier than monomers (green box) were enriched for CD1d-TCR dimers, as confirmed by polyacrylamide gel electrophoresis, and were pooled to optimise lipids entering mass spectrometry experiments (MS). Fractions were normalised to protein input and then subjected to lipid elution. The eluents were analysed by the negative mode nano-electrospray (middle) and HPLC–Quadruple Time of Flight–MS (right) to identify ions and mass chromatograms identifiable as SMs (red) and PCs (blue) with the indicated m/z values, total chain length (C) and unsaturations (0, 1, 2). Data is representative of *n* = *3* independent experiments involving T26A. **(b)** The same analysis for CD1d-T26B, CD1d-T14, and CD1d-VD3G8 TCR complexes was performed for *n = 1,* *n = 1* and *n = 2* trap experiments, respectively. (**c**) TRAV26^+^ (T26A, T26B) and TRAV26^-^ (T14, VD3G8) CD1d-endo-reactive type II NKT TCR-transduced SKW3.β2m^−/−^ cell lines, were stained with CD1d-tetramers, each exogenously treated with a different ligand. The type I NKT15 TCR and the ClPPBF-reactive type II VD1G9 TCR clones were included as controls. The fold variation in CD1d tetramer mean fluorescence intensity (MFI) ± SEM, relative to CD1d-endo is shown, from *n* = *3* independent experiments, each represented by a single point, except for VD3G8, as well as T14 staining with CD1d-LPE, and any conditions involving CD1d-SM42:2 where *n* = 2.

Two hypotheses regarding the role of lipids in CD1d-TCR binding were investigated. The first was that TRAV26^+^ TCRs bind CD1d in a lipid-independent fashion, reminiscent of what has been reported for CD1a^37,38^ and CD1c^39^. The second, supported by studies of known type II NKT cell ligands, sulfatides^40^ and membrane phospholipids^41^, was that some as yet unknown but ubiquitous self-lipid can increase the TCR binding-affinity to CD1d and that α-GalCer can substitute for this lipid. To test these hypotheses, the ‘TCR trap’ method^42,43^ was employed whereby the soluble TCRs were allowed to form a complex with CD1d-endo that was subsequently purified using size exclusion chromatography, where TCR-bound CD1d molecules can be separated from non-bound CD1d molecules (Fig. 3a). The eluents of CD1d-TCR were then compared to CD1d alone by mass spectrometry analysis to identify the lipids specifically associated with CD1d-TCR binding.

The human CD1d-endo carries a heterogeneous cargo of self-lipids that are detectable by nano-electrospray and reversed phase high performance liquid chromatography-mass spectrometry (Fig. 3a)^42,43^. Nano-electrospray analysis was conducted on lipids that were eluted from the TCR alone, CD1d alone and CD1d in complex with the two TRAV26^+^ αβ TCRs (T26A and T26B), the γδ TCR (VD3G8) and the TRAV26^–^ αβ TCR (T14) (Fig. 3b). For TCR alone no clear signals for lipid binding were observed, as expected (Fig. 3a). In contrast, for CD1d alone, there were many *m/z* signals in negative mode. Similar to recent results with CD1a^38^, and other CD1 molecules^30^ the strongest signals matched the absolute mass and alkane series patterns for sphingomyelins (SM, *m/z* 737.6, 765.6, 819.6, 845.6 and 847.6) and some weak signals matched for phosphatidylcholine (PC, *m/z* 794.6, 904.7, 906.7, 932.7 and 934.7), which are the two most abundant cellular sphingolipid and phospholipid classes, respectively. In contrast to results from a type I NKT TCR-trap study in which the TCR strongly influenced the lipids bound to CD1d^43^, CD1d in complex with T26A, T26B, VD3G8 and T14 showed a lipid pattern that was highly similar to CD1d alone. Using HPLC-MS as a more quantitative method that separates individual molecules for detection in very narrow mass windows, once again the distinct nature of each TCR in the four types of CD1d-TCR complexes, did not substantially affect the repertoire of lipids that mediated its binding to CD1d^43^. Indeed, in all cases, the patterns of lipids in CD1d-TCR complexes matched the patterns of lipids seen for CD1d-endo alone (Figs. 3a and 3b), maintaining high SM to PC ratio^30^. Thus, no particular exogenous lipid was required for CD1d-TRAV26^+^ TCR complex formation, supportive of the first hypothesis that these TCRs bind in Ag-independent manner.

To independently assess reactivity towards individual lipid species that include known CD1d-presented Ags that might be under-represented or absent from the mixed cargo of endogenous lipids, (dominated by SMs and PCs that were incorporated into CD1d during its biosynthesis in GnTI^−/−^ HEK293S cells^30^, Fig. 3a–b), we generated a panel of CD1d tetramers by exogenously loading secreted CD1d with one of the following CD1d-Ags or controls: phosphatidylethanolamine (PE), phosphatidylinositol (PI), phosphatidylcholine (PC), phosphatidylserine (PS), sphingomyelin (SM C34:1 or C42:2), sulfatide, lyso-(L)PE, LPC, phosphatidylglycerol (PG), ganglioside GD3, α-GalCer, 3-chlorophenyl-2,2,4,6,7-pentamethyldihydrobenzofuran-5-sulfonate (ClPPBF)^7^ or vehicle (tyloxapol) (Fig. 3c). Each tetramer was tested for staining the type II NKT TCR reporter lines (VD3G8, T14, T26A, T26B) and the control NKT lines that expressed the type I NKT15 TCR and the ClPPBF-reactive type II VD1G9 TCR^7^ (Fig. 3c). NKT15 and VD1G9 stained only with tetramers carrying their known cognate Ags and not with CD1d-endo. In contrast, amongst all type II NKT lines that were stained with CD1d-endo tetramers (Fig. 2d), T26A and T26B bound CD1d tetramers at similar intensities, regardless of the ligand exogenously added (Fig. 3c**)**, including the bulky GD3 glycolipid^44^, whereas T14 and VD3G8 stained less with CD1d-α-GalCer tetramers (Fig. 3c), and in line with Fig. 2d. Thus, in contrast to other NKT lines tested, lipid replacement within CD1d did not markedly affect CD1d-TRAV26 TCR interactions in these systems.

### CD1d binding affinity of type II NKT TCRs

**Fig. 4:**
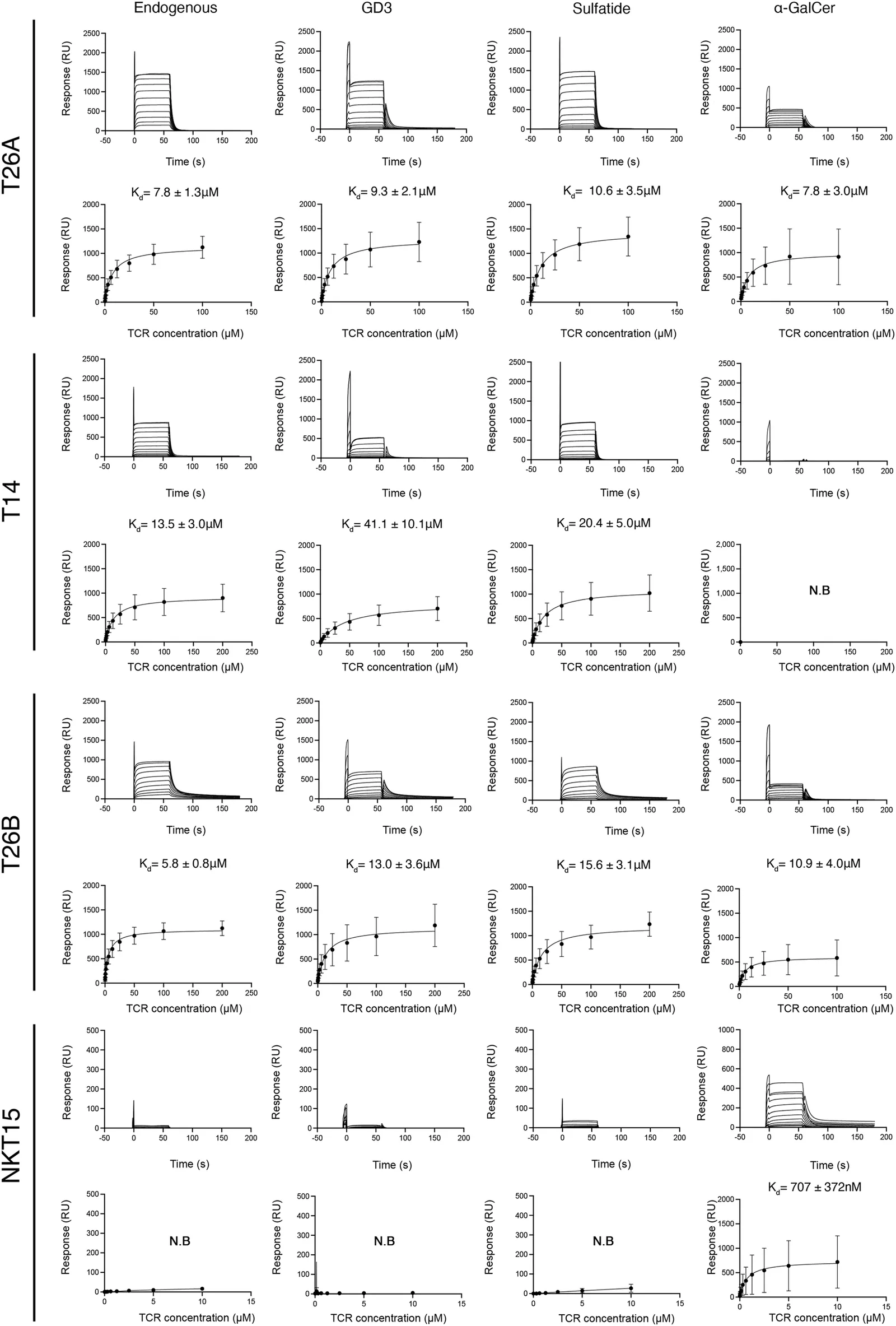
Affinity measurements of type II NKT TCRs. Surface plasmon resonance (SPR) of soluble T14, T26A, T26B and NKT15 NKT TCRs against CD1d-endo, CD1d-GD3, CD1d-sulfatide and CD1d-α-GalCer. The shown sensograms are representative of one experiment performed in duplicate. The resulting Kd ± SEM values are derived from *n* = *6* independent experiments (T26A, T26B and T14 TCRs) and *n* = *3* (NKT15 TCR) and each performed in duplicate. Error bars in the regression curves indicate mean ± SD.

Using surface plasmon resonance (SPR), the affinity of the interaction between three soluble type II NKT TCRs towards CD1d-endo and CD1d bearing sulfatide, GD3 or α-GalCer was examined. The T26A TCR bound to CD1d-endo with an affinity of ~7.8 μM and this was not impacted by the type of lipid associated with CD1d, with *K*_D_ values ranging from 7.8 μM to 10.6 μM (Fig. 4). Similarly, the T26B TCR bound to CD1d-endo or CD1d with each of the three defined lipids with a similar range of affinities (5.8-15.6 μM) (Fig. 4). Conversely, the T14 TCR exhibited a finer specificity to the lipids presented by CD1d (Fig. 4). Here, whilst the T14 TCR bound to CD1d-endo, CD1d-sulfatide and CD1d-GD3 with *K*_D_ values of 13.5, 20.4 and 41.1 μM, respectively, it did not bind to CD1d-α-GalCer (*K*_D_ > 200 μM), in agreement with the lack of CD1d-α-GalCer tetramer staining of this line (Fig. 2d**)**. Furthermore, none of the tested ligands, including sulfatide, GD3, SM or α-GalCer, enhanced activation of the type II NKT TCR-transduced cell lines measured by CD69 upregulation, whereas α-GalCer elicited stronger activation of the NKT15 type I NKT TCR line, over that elicited by C1R.CD1d or C1R.WT cells (Fig. S3a). These responses reflected specific NKT TCR-CD1d interactions, as they were blocked by anti-CD1d, whereas the control AF7 MAIT TCR^45,46^ expressing cells only activated in the presence of 5-OP-RU (Fig. S3b). Collectively, these results suggest that the TRAV26^+^ TCRs interact with CD1d with similar affinity irrespective of the lipid bound.

### Structure of the TRAV26^+^ type II NKT TCR-CD1d-lipid complex

**Fig. 5:**
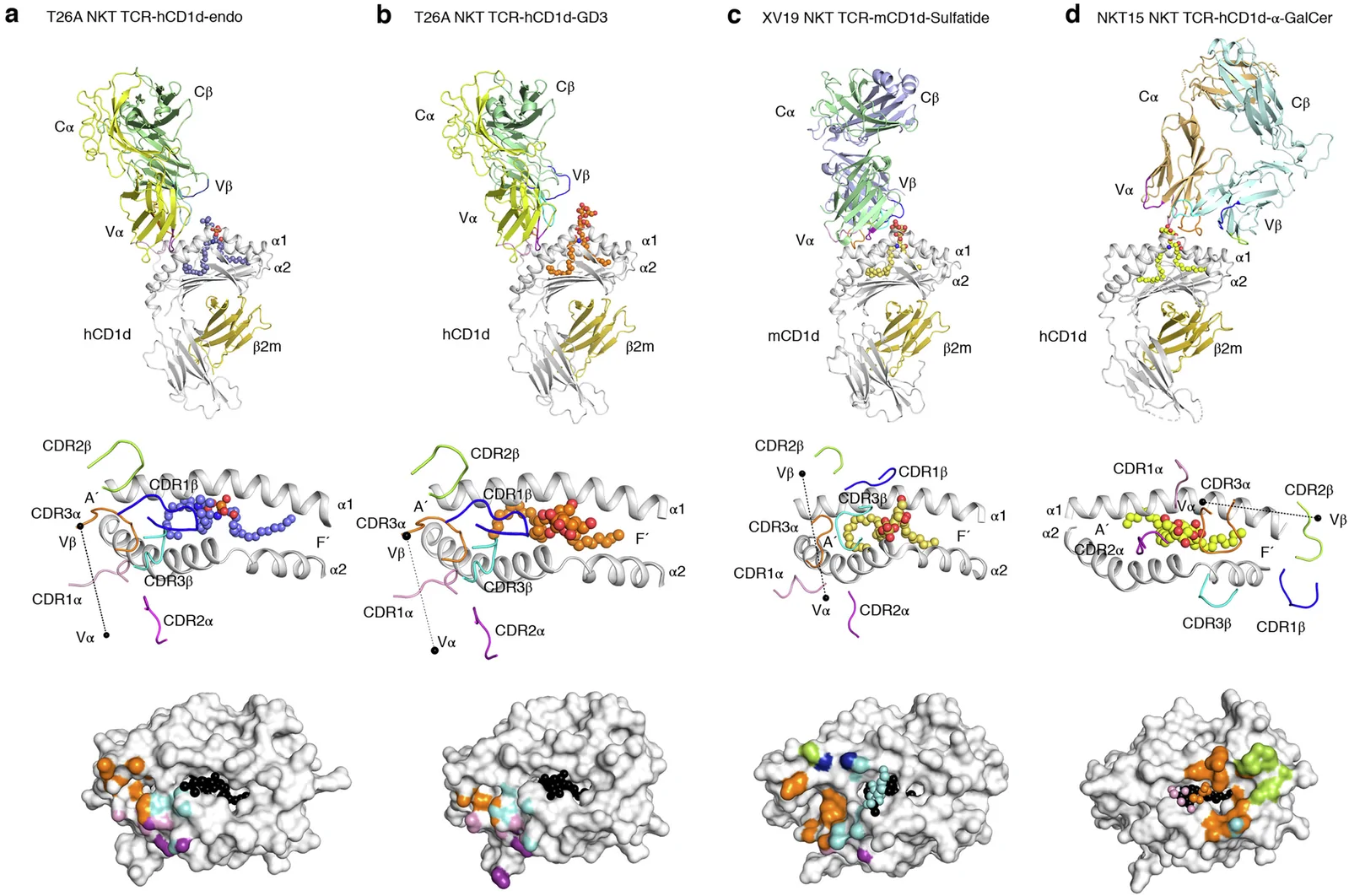
Overview of the mouse type II XV19, human type II T26A and human type I NKT15 NKT TCRs-CD1d ternary complexes. Crystal structure of the **(a)** human T26A TCR-CD1d-endo (SM C40:2 modelled), **(b)** T26A TCR-CD1d-GD3, **(c)** mouse XV19 TCR-CD1d-α-GalCer (PDB code: 4EI5) and **(d)** human NKT15 TCR-CD1d-α-GalCer (PDB code: 2PO6) ternary complexes. The top panels show a cartoon representation of each ternary complex, middle panels depict a top view of the CD1d-binding cleft and the bottom panels illustrate the TCR footprints on the CD1d-lipid molecular surface. Top panels: the CD1d and β2-microglobulin (β2m) molecules are coloured in grey and gold, respectively. XV19 TCRα, light green; XV19 TCRβ, light purple; T26A TCRα, yellow; T26A TCRβ, green; NKT15 TCRα, light brown; NKT15 TCRβ, light blue. The CDR loops are coloured as follows: CDR1α, pink; CDR2α, magenta; CDR3α, orange; CDR1β, blue; CDR2β, lemon green; CDR3β, cyan. The carbon atoms of the lipids, sphingomyelin (SM 40:2), GD3, sulfatide and α-GalCer are coloured violet, orange, pale yellow and yellow spheres, respectively. The oxygen and nitrogen atoms are coloured red and blue, respectively. Middle panels: the lipids are shown as spheres and coloured as in top panels. The centre of mass of the respective TRAV and TRBV variable domains are shown as black spheres. Bottom panels: the molecular surface of CD1d is coloured in light grey. Lipids are in black. CDRs TCR contact sites are coloured as in top panels.

We determined the crystal structure of the T26A NKT TCR-CD1d-endo ternary complex (Fig. 5a and Table S1). The T26A NKT TCR docked ~100° across the main axis of the CD1d Ag-binding cleft, being positioned over the ‘left’ side of the platform on the A’-roof of CD1d, sitting closer to the α_2_-helix (Fig. 5a). This docking position clearly contrasted to type I NKT TCR-CD1d-lipid binding (Fig. 5d). This ‘left-shifted’ binding footprint was more reminiscent of the recognition strategy adopted by CD1a autoreactive TCRs^3,42^, CD1d-sulfatide-reactive type II NKT TCRs^19,20^ (Fig. 5c) and some ‘atypical’ human NKT TCRs^22–26^ whereby the TCRs contacted CD1d and the lipid presented.

**Fig. 6:**
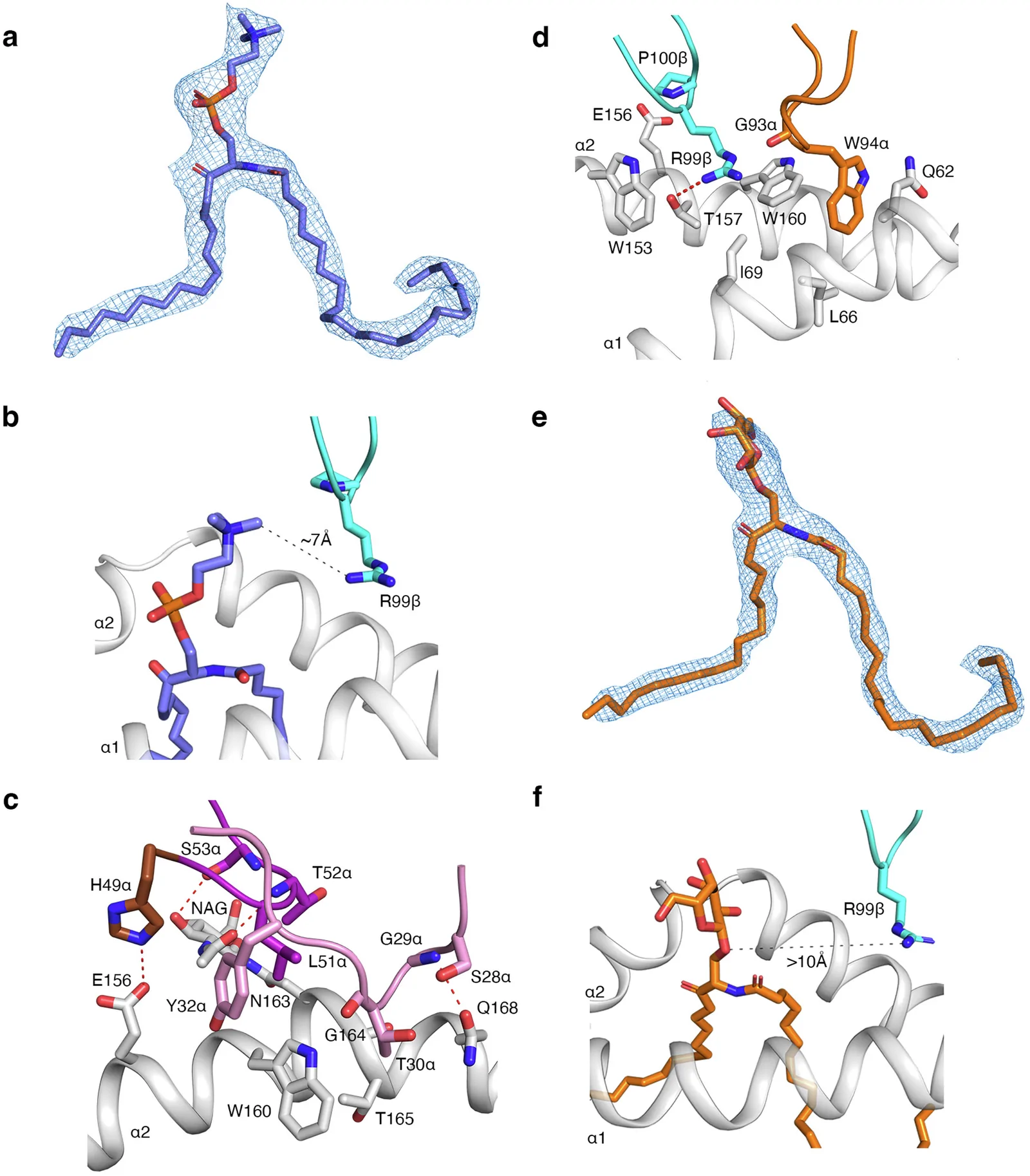
Molecular interactions at the T26A TCR/CD1d-SM. **(a)** 2Fo-Fc electron density map of SM contoured at 0.8σ level. SM is shown as blue sticks. **(b-d)** Molecular interactions of the T26A CDR1α (pink), CDR2α (purple), CDR3α (orange), CDR3β (cyan) and FWα (brown) with CD1d-SM. **(e)** 2Fo-Fc electron density map of GD3 (partially modelled) contoured at 0.8σ level with GD3 shown as orange sticks. **(f)** Molecular interactions of T26A TCR with GD3. For clarity, the α1- and α2-helices of CD1d are shown as cartoon representation and coloured in light grey.

Mass spectrometry analysis of the T26A TCR-CD1d-endo demonstrated that although mixed lipids were bound, long chain SM ( > C40) appeared to be the major species present (Fig. 3a**)**. In line with this, we modelled and refined SM (C40:2) as the bound endogenous lipid into the unbiased electron density that was clearly visible within the CD1 cleft (Fig. 6a and Fig. S2a). The T26A TCR did not directly contact the protruding headgroup of the lipid bound within the CD1d cleft whereby the most proximal residue to SM was located > 7 Å away (Fig. 6b**)**. This distal mode of binding can explain why these TCRs seemingly bind to CD1d in a lipid-independent manner and indeed tolerate binding to CD1d carrying lipids with head groups that differ in size and shape (Figs. 3 and 4). Thus, the TRAV26A^+^ NKT TCR contacts CD1d itself, which breaks the CD1d-lipid co-recognition paradigm.

### Structural basis of TCRα chain bias towards CD1d

Upon complexation, the buried surface area (BSA) of the T26A NKT TCR-CD1d-endo complex ( ~ 600 Å^2^), fell outside the range for type I NKT TCR-CD1d ternary complexes (760-860 Å^2^) and was significantly lower than the mouse type II NKT TCR-CD1d-lipid complexes ( ~ 1000 Å^2^)^19–21^. The TCRα and TCRβ chains of T26A contributed ~75% and ~25% of the total BSA at the TCR-CD1d interface, respectively, with both the CDR1α (21% BSA) and CDR2α (16% BSA) loops contributing to the TCR-CD1d interface thereby providing immediate insight into the TRAV26 bias. Conversely, the CDR1β and CDR2β did not interact with the CD1d-endo complex, explaining the broader observation of TRBV gene diversity amongst TRAV26 TCRs (Table 2).

The CDR1α loop sat peripherally above the α2-helix of CD1d whereby Thr30α interacted with Trp160, Gly164, Thr165 and Gln168 residues of CD1d, while Tyr32α stacked against Glu156 and Trp160 (Fig. 6c and Table S2). Ser28α contacted only Gln168 while Gly29α contacted Gly164 and Gln168 of CD1d via van der Waals (VDW) and hydrogen bond interactions, respectively (Fig. 6c and Table S2). Within the CDR2α loop, Leu51α, Thr52α and Ser53α contacted Asn163 of CD1d and its N-linked glycans (N-acetylglucosamine, NAG) (Fig. 6c and Table S2). Further, the germline encoded framework His49α that precedes the CDR2α loop established a VDW interaction with Glu156 in CD1d (Table S2). Interestingly, Thr30α, Thr52α and Ser53α mediated several contact points with CD1d and were not conserved in *TRAV26-1* genes, providing molecular insight for why *TRAV26-2* may be over-represented between the two genes amongst CD1d-endo-reactive cells (Table 2).

The CDR3α and CDR3β loops dominated the interactions at the interface with 35% and 25% BSA, respectively (Fig. 6c, d). The CDR3α and CDR3β loops were positioned more centrally over the CD1d binding-cleft. Here, Arg99β from the CDR3β loop plunged into the CD1d-binding cleft to form VDW interactions with Trp153, Thr157 and Trp160, while Pro100β contacted Glu156 of CD1d (Fig. 6b). The non-germline encoded Gly-Trp motif, which is conserved in 6 out of the 39 TRAV26-2 clones from 4 distinct donors (Fig. 2b and Table 2), established multiple contacts with CD1d (Fig. 6d and Table S2). Whereas in 18 of the 39 TRAV26-2 clones, a Phe residue was present at this position, which constitutes a Gly-Phe motif (Fig. 2b and Table 2). Interestingly, this Phe residue was germline-encoded in 7 of the TRAV26-2-TRAJ48^+^ clones across 6 donors. The other 11 clones harbouring the Gly-Phe motif were all non-germline encoded (Fig. 2b and Table 2). Conservation of bulky aromatic residues within the CDR3α across 74% of the TRAV26-2^+^ clones may be a key structural determinant governing the TCR docking topology. Further, bias towards Phe may also explain the relatively high occurrence of *TRAV26-2–TRAJ48* rearrangements. The G93α residue was also highly prevalent in both motifs, which might be preferentially selected over other larger amino acids to avoid steric clashes with Trp160 and/or CDR3β residues (Fig. 6d). This finding aligned with the additional patterns observed for TRAV26-2 isolated sequences in positions 6 and 7 (Fig. 2b bottom) and emphasised the need for a bulky residue in position 7 (Fig. 2b). Collectively, these results suggested that the germline residues encoded by the *TRAV26-2* gene, and the rearrangement of non-germline-encoded structural motifs to Gly-Phe/Trp, played a key role in determining the docking modality adopted by the T26A TCR over the α2-helix A′-roof of CD1d.

### A common mode of recognition of self-lipids by the T26A NKT TCR

Structural studies on the T26A TCR-CD1d-lipid recognition were extended by loading GD3 into CD1d (Fig. S2b**)** and determining the crystal structure of the ternary complex (Fig. 5). The electron density for the bound GD3 was partially visible whereby only the first glucose moiety of the headgroup and both lipid tails could be modelled (Fig. 6e and Fig. S2c). The crystal structure revealed a nearly identical docking mode to that of CD1d-endo complex that was modelled as CD1d-SM (Fig. 5). The majority of the molecular interactions at the T26A NKT TCR-CD1d-GD3 interface were also conserved (Fig. S2d-e and Table S3). Indeed, the T26A NKT TCR did not contact the GD3 lipid, as the most proximal TCR residue, Arg99β from the CDR3β, was 10 Å away from the glucose moiety (Fig. 6f). Other sphingolipid Ags such as α-GalCer would be expected to produce similar observations (Fig. S2f). Collectively, our structural analysis of the T26A type II NKT TCR introduces a molecular paradigm for CD1d recognition whereby the type II TRAV26^+^ NKT TCR can bind CD1d in a lipid-independent manner.

### Molecular footprint of TRAV26 type II NKT TCRs onto CD1d

**Fig. 7:**
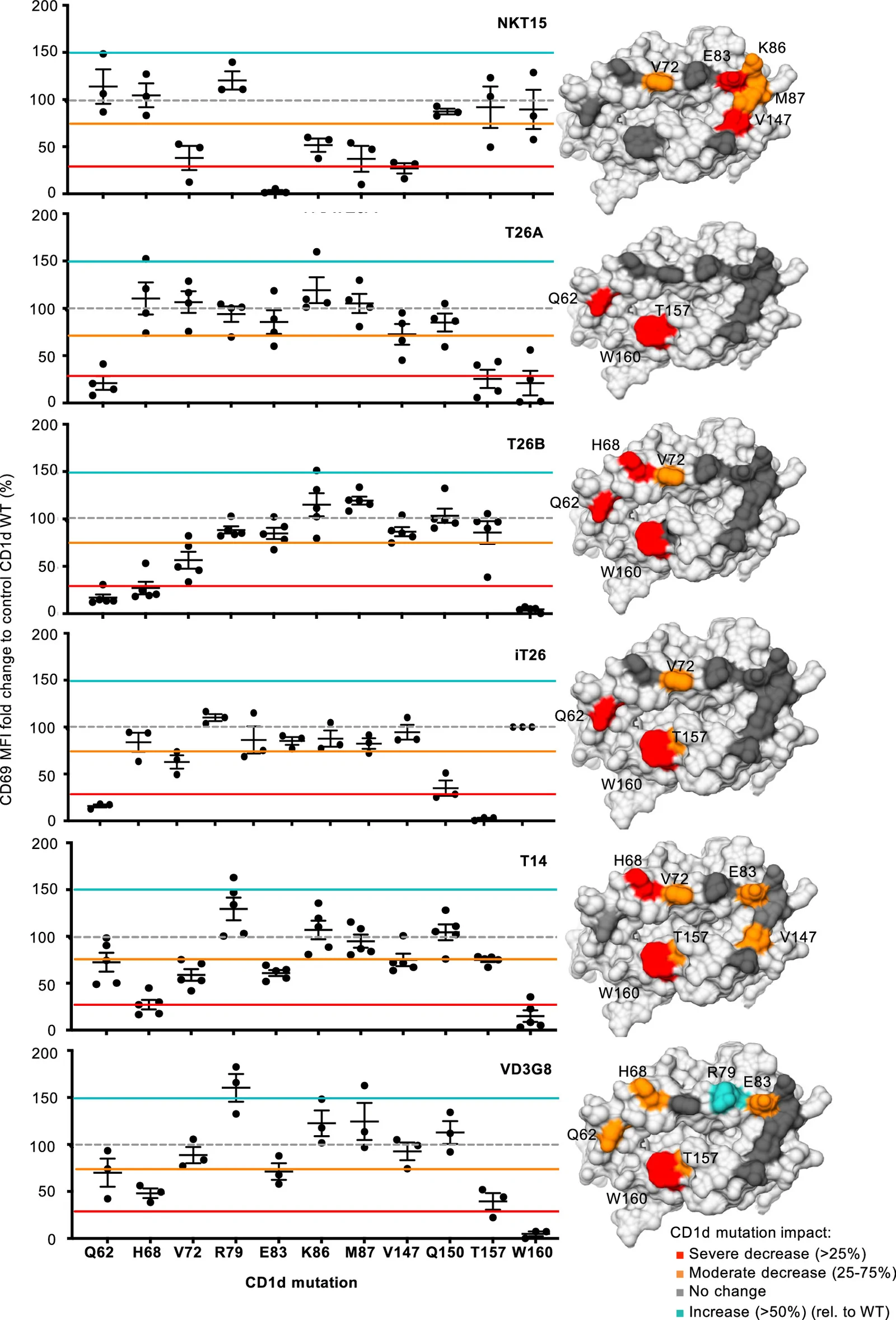
CD1d docking modes of CD1d-endo reactive type II NKT cells. TRAV26^+^ (T26A, T26B and iT26), and TRAV26^-^ (T14 and VD3G8) CD1d-endo reactive NKT TCR-transduced SKW3.β2m^−/−^ cell lines were co-cultured for 16 h with C1R cells each transduced with a single (Ala) mutated version of CD1d. The responses by the type I NKT15 TCR-expressing cell line were analysed as a control. The level of activation (CD69-fold change in comparison to TCR-transduced cell lines alone) elicited by each mutant line is normalised to the response elicited to *wild type* (WT) C1R.CD1d. Data depict mean ± SEM from *n* = 3 independent experiments for NKT15, T26A and iT26, *n* = 4 for T26A, T26B and *n* = 5 for T14. Corresponding CD1d molecular surface maps (Protein Data Bank code: LZT4) are shown to the right of each graph, depicting residues that, when mutated, had no effect (dark grey), a minor ( < 25%, red), major ( < 75%, orange) fold decrease, or a fold increase ( > 50%, turquoise) in CD69 MFI relative to WT CD1d.

Having established that lipid Ags had little impact on TRAV26^+^ TCRs binding to CD1d, the next step was to assess their functional CD1d docking footprints. Here, C1R cell lines overexpressing wild type CD1d or alanine-substituted CD1d mutants targeting solvent-exposed residues of CD1d^7^ were used to test their ability to stimulate the TRAV26^+^ and TRAV26^–^ TCR-transduced cell lines (Fig. 7). CD69 upregulation on the NKT15 TCR control line was inhibited by the alanine mutations of Glu83, Val147, Lys86, and Met87, all positioned near to the F’-portal of CD1d, consistent with our previous results^7^ and the ‘right-shifted’ parallel docking strategy adopted by type I NKT TCR(s)^35^ (Fig. 7). In contrast, the three TRAV26^+^ TCR transduced cell lines were not impacted by those mutations but were inhibited ( > 75%) by mutations of two CD1d residues Gln62 and Trp160, located distant from the F′-portal, where Ags protrude. These data complement the structural data showing that TRAV26^+^ TCRs bind the A′-roof. The impact of the CD1d alanine mutant scan was similar, but not identical, across the three TRAV26 TCR lines. This outcome likely reflects differences in CDR3α and TCRβ chain pairing, and their influence on CD1d-binding.

Activation of both TRAV26^–^ TCR transduced lines (T14 and VD3G8) was severely impacted by mutations of Trp160 and His68, which sit in the A’-roof of CD1d, in addition to other residues closer to the Ag portal, spanning to the F’-roof. Specifically, T14 was moderately inhibited by mutations of Val72, Thr157, Glu83 and Val147 and VD3G8 impacted by Thr157, Glu83, whereas Arg79Ala mutation led to enhanced activation, suggesting that Arg79 interferes with optimal VD3G8 TCR binding to CD1d. Thus, these TRAV26^–^ TCRs appeared to dock more centrally and over the Ag portal, which likely reflects their differences in sensing CD1d-bound ligands (Figs. 2c, 3c and 4), unlike the extreme A’-roof CD1d-docking by TRAV26^+^ TCRs. Collectively, these data suggest that TRAV26^+^ NKT TCRs bind to CD1d in a left-shifted approach and ligand-independent manner.

## Discussion

Currently, the CD1d-restricted repertoire is thought to comprise a highly conserved population of type I NKT cells, and a group of diverse type II NKT cells. Here, we identify a public population of type II NKT cells defined by a TRAV26 TCR bias and a conserved CDR3α motif and we provide detailed molecular insight into their mode of lipid-independent CD1d recognition. With wider implementation of CD1 tetramers to systematically investigate TCR repertoires, we are increasingly detecting conserved patterns and public TCR usage for particular CD1-Ag pairs^7,47,48^^,^^49^^,^^50^^,^. A general view emerging from these studies is that NKT cells are not exclusively represented by a public TCR, but instead that non-polymorphic CD1 proteins generate an underlying network of T cells shared across donors, with many conserved families of TCRs and Ags.

The epitope for the TRAV26 TCR is CD1d itself rather than a defined lipid. Thus, an exposed A’-roof is likely a common or nearly universal epitope in humans, which could account for the several distinct TRAV26-TRAJ48^+^ T cell clones seen in every donor tested here. These data connect to a prior study where two distinct *TRAV26-2* encoded TCRs (*TRAV26-2—TRAJ48* and *TRAV26-2—TRAJ44*) were reported among 14 *TRAV10*^*-*^*TRAJ18*^*-*^*TRBV25*^*-*^ TCRs isolated with CD1d-α-GalCer tetramers^25^, albeit not recognised as a conserved motif through the strategy employed at the time. The Ag-independent mode of CD1d recognition by germline-encoded TRAV26 TCR residues and a conserved CDR3α motif raises interesting questions about the evolutionary pressures that lead to the conservation of TCRs to act as pattern recognition receptors against CD1d.

Thus, our results also suggest that some type II NKT cells might be poised to sense overall changes in surface expression of the Ag-presenting molecule CD1d, rather than the lipid-Ag itself, during inflammatory or disease settings. This is illustrated by activation, albeit modest, of a TRAV26^+^ line following increases in CD1d surface expression of in vitro differentiated MoDCs, compared to primary CD14^+^ monocytes. Accordingly, changes in surface expression levels of CD1d have been shown to impact NKT cell function^51^ and are often associated with infection^52–56^, cancer^57^ and autoimmunity^58^. For example, hepatocytes that are chronically infected with hepatitis C virus display high CD1d surface expression relative to healthy tissue or other liver disease, to levels that resemble those of C1R.CD1d transfectants that elicit IFN-γ release from intrahepatic CD1d-reactive T cells (type II NKT)^59^. Similarly, another study demonstrated that CD1d overexpression can be targeted by mouse CD1d-reactive Vγ4^+^ T cells, promoting viral myocarditis *sequela* of coxsackievirus infections^60^. Whether these physiological contexts can lead to activation of TRAV26^+^ NKT cells is an important question for further exploration.

Whilst some type II NKT clones can display lipid-Ag specificity^19–21,61,62^ and mount lipid Ag-driven responses^12,13,63^, other type II NKT cells also retain the ability to recognise CD1d carrying multiple self-Ags^4,27,28,61^ possibly as a strategy to sense early stress signals in an innate-like fashion^56,64–66^. Self-reactive profiles have been reported amongst other CD1-restricted lipid-reactive T cells in humans^2^ and ligand-independent interactions have been described for CD1a^38,67^, CD1b^68^, CD1c^39^ and CD1d^69^, and MR1^70^, where the structural mechanism in part relies on smaller Ags hiding inside antigen-binding clefts (particularly for the CD1 system)^3^. However, non-permissive lipids, such as long chain SM (42:2), bound by CD1a or CD1c can prevent docking of endo-reactive TCRs^39,42,71^. Here, we show that TRAV26 TCRs engage CD1d when displaying a variety of headgroup-containing lipids, over the extreme side of the A´-roof, without contacting the protruding headgroups. Accordingly, both the α1 and α2 helixes of the A´-roof are heavily contacted by TRAV26-2 TCR α-chain and may form an anchor region within CD1d targeted by non-type I NKT TCRs, including the ‘atypical’ CD1d-α-GalCer reactive NKT TCRs (*TRAV10–TRAJ18*^*−*^)^22–26^, CD1d-benzofuran-self-lipid reactive type II NKT TCRs^7^, and the murine sulfatide reactive TCRs^19,20^. However, all previously structurally described CD1d interactions involve NKT TCR co-engagement of CD1d and their cognate lipid, which contrasts our study of TRAV26^+^ TCRs.

The TCR patterns identified among CD1d-endo reactive clones followed in vitro expansion of sorted cells, which is commonly used in studies of rare populations^7,22,24,32^ and was necessary for us to confidently identify these cells. While it is possible this may have altered the representation of some TCRs within the population, the expansion is based on anti-CD3 stimulation and therefore should not inherently favour any particular TCRs. While this approach precludes addressing immune-function ex vivo, nonetheless, we have shown that expanded CD1d-endo tetramer^+^ cells, including TRAV26 cells, express PLZF and are capable of producing a range of cytokines, in particular TNF and IFNγ, implying that these cells share innate-like T cell features and have pro-inflammatory potential. Future studies will investigate their contributions to various health and disease states. It will also be important to address the intrinsic or extrinsic regulatory mechanisms that define the activity and immune function of TRAV26^+^ type II NKT cells, including their reliance on secondary signals such as cytokines^66^ or other receptors such as NK receptors^72^, which can play a role in modulating activation status in both TCR-dependent and TCR-independent manners. Most importantly, our findings suggest that TRAV26 cells are highly represented within the CD1d-endo reactive population and are present as a public TCR in essentially all individuals tested. The potential significance of these observations is highlighted by an earlier study where we revealed the TRAV12-TRAJ6 type II NKT TCR motif^7^, specifically reactive to small benzofuran-like molecules bound by CD1d. That TCR motif was subsequently implicated in Crohn’s disease^73,74^ where the cells were named Crohn’s-associated invariant T cells (CAIT cells), which is now underpinning studies to investigate the contributions of CAIT cells and the CD1d axis to this disease.

Several candidate therapies have explored the immunomodulatory potential of type I NKT cells, with ongoing trials also including CAR-type I NKT cell allogenic and adoptive therapies^75^. The findings of this study may offer new avenues to modulate immune responses through the monomorphic protein CD1d and overcome existing limitations of current trials, that require α-GalCer co-delivery to allow type I NKT-activation^57^. Moreover, our observation that TRAV26 TCRs retain their ability to recognise the complex glycan headgroup of GD3 loaded onto CD1d, which inhibits type I NKT responses^44^, flags their potential exploration in therapies against GD3-expressing cancers. These findings further highlight the possibility of future immunomodulation agonists, antagonists or anti-TCR antibodies to selectively manipulate these public TRAV26^+^ type II NKT cells.

## Methods

### Flow cytometry

Buffy coats from healthy blood donors were obtained from the Australian Red Cross Blood Service (agreement 13-04VIC-07, 22-11VIC-04 and 24-10VIC-15), and all experiments were conducted in accordance with the University of Melbourne Human Research and Ethics Committee guidelines (approval numbers 1035100, 13000 and 30058). PBMCs were prepared through density gradient centrifugation using Ficoll-Paque (GE Healthcare). Cells were incubated for 10 min with Fc-receptor block (BD Pharmingen) and either 5% (v/v) mouse serum or anti-CD36 (5-271, Biolegend), followed by CD1d-endo tetramers for 30 min on ice. Where described, CD1d–endo tetramer^+^ cells were enriched by TAME, using anti-phycoerythrin (PE) magnetic beads and LS columns (Miltenyi Biotec), and stained with secondary antibodies (Table S4) including: CD3ε (UCHT1, eBioscience and Becton Dickinson), CD4 (RPA-T4, BD Pharmingen), CD8α (SK1, BD Pharmingen), CD19 (HIB19, BioLegend), CD14 (MφP9, BD Pharmingen), TCRγδ (11F2, BD Pharmingen), CD42b (HIP1, BD Pharmingen) and 7-aminoactinomycin D (7AAD) viability dye (Sigma), or Live/Dead Fixable Near-IR Dead Cell Stain (Thermo Scientific). CD3^+^ CD1d–endo tetramer^+^ cells were sorted using a FACSAria (BD Biosciences). When reassessed immediately after the first-sort, purities ranged 13–65%. Where described, sorted cells were then expanded for 18–21 days, whereby in the first two they were exposed to plate bound anti-CD3 (10 µg/mL, UCHT1, BD Pharmingen) and anti-CD28 (2 µg/mL, CD28.2, BD Pharmingen), in the presence of IL-2 (20 U/mL, Preprotech) IL-7 (50 ng/mL, eBioscience) and IL-15 (40 pg/mL), in complete RPMI-1640 supplemented with 10% (v/v) FBS (JRH Biosciences), penicillin (100 U/ml, Sigma), streptomycin (100 μg/ml), Glutamax (2 mM), sodium pyruvate (1 mM), nonessential amino acids (0.1 mM), HEPES buffer (15 mM, pH 7.2–7.5) (all from Invitrogen, Life Technologies) and 2-mercaptoethanol (50 μM, Sigma). After expansion, cells were re-stained with CD1d-endo tetramers alone, followed by surface antibodies and in some instances secondary CD1d-α-GalCer tetramer staining. These sorted and expanded CD3^+^CD1d-endo tetramer^+^ cells were then index-sorted as single cells for TCR sequencing. Transcription factor intracellular staining was performed using Foxp3/Transcription Factor Staining Buffer Set (BD Biosciences) and anti-PLZF (R17-809 BD Horizon). Data analysis was completed using FlowJo 10 (BD), and graphs were generated using GraphPad Prism 10.

### Lipids

α-GalCer C24:1 (PBS44) used for CD1d tetramers was kindly provided by P. Savage (Brigham Young University). α-GalCer C26:0 used for cellular activation assays was supplied by Alexis Biochemicals, and SM C42:2 (24:1/18:1), SM C34:1 (16:0/18:1), PC C42:1; sulfatide C42:2, PE C36:1, PS C36:1 PC C36:1, PC C42:2, PI C36:1, PG C36:1, LPE C18:1, LPC C18:1, pLPE C18:1 from Avanti Polar Lipids. Disialo-ganglioside GD3 (mixture containing C40:1, C41:1 and C42:1) was purchased from Matreya. CD1d-ligands were dissolved in tris buffer saline (TBS) alone (pH 8) or TBS containing 0.05% v/v tyloxapol (Sigma), or buffer containing 0.5% v/v tween-20, 57 mg/ml sucrose and 7.5 mg/ml histidine and exogenously loaded overnight into soluble biotinylated CD1d at 12-fold molar excess (except for α-GalCer - loaded at 6-fold), prior to tetramerization.

### Identification of the NKT TCRs

CD3^+^CD1d-endo tetramer^+^ cells were single-cell sorted from CD1d-endo tetramer-enriched and culture-expanded NKT cells (as described above), and cDNA generated using 0.1% Triton X-100 (Invitrogen) and SuperScript VILO according to manufacturer’s instructions. Paired TCRα and TCRβ or TCRγ and TCRδ chain-transcripts were amplified by two rounds of multiplex nested PCR with a panel of Vα and Vβ or Vγ and Vδ primers (Table S5) and GoTaq Master Mix (Promega), as previously described^31^. PCR products were separated on a 1.5% agarose gel, purified and sequenced at the AGRF facility (Australia) and analysed using IMGT^76^. TCR nomenclature is presented in accordance with the IMGT guidelines. Unproductive TCR gene rearrangements were excluded from analysis. The first 10 amino acids of the CDR3α of TRAV26 TCRs shown in Fig. 2b were aligned using Logomaker^77^.

### Generation of stable cell lines and stimulation assay

TCR constructs containing the full-length TCRα and TCRβ or TCRδ and TCRγ chains separated by a 2A-cleavable linker were synthesised (Genscript) and cloned into the pMIGII plasmid. TCR-deficient SKW3.β2m^−/−^ cells were retrovirally transduced with the TCR and a 2A-cleavable human CD3^78^, plus the packaging vectors pEQ-Pam-3-E pVSV-G and using HEK293T cells as packaging cells and FUGENE 6 (Promega), as previously described^79^. The pMIGII, expression and packaging vectors were provided by Dr. Dario Vignali (St. Jude’s Research Hospital, USA), and the CD3 expression vector was provided by Prof. Stephen Turner (Monash University, Australia). CD3(GFP)^hi^ cells were sorted and assessed for their ability to bind CD1d tetramers by flow cytometry. For Fig. 2C, HEK293T cells were transiently transduced with CD3 and TCR vectors in the same manner but in the absence of packaging vectors and assessed after 48 h for CD3/TCR expression and CD1d-tetramer binding by flow cytometry. C1R cells were transduced to express human CD1d or mutated versions of CD1d, akin to SKW3.β2m^−/−^ cells, and purified using flow cytometric sorting to produce stable cell lines expressing similar surface levels of CD1d^7^.

### Activation assays

For stimulation assays, TCR-expressing SKW3.β2m^−/−^ cells were co-cultured overnight in complete RPMI (as described above), with or without C1R cells (either C1R.WT, C1R.CD1d or C1R.β2m^−/−^) cells, with α-GalCer C26:0 at 1 μg/mL, sulfatide C42:2, SM C42:2 or GD3 (mixture containing C40:1, C41:1 and C42:1) at 20 μg/mL, or vehicle controls, and with either anti-CD1d (51.1, Biolegend), isotype control antibody (MPC-11 or MG2b-57, Biolegend) or media alone, in round-bottom 96-well plates. Activation of TCR-transduced cells was analysed by flow cytometry for CD69 upregulation using anti-CD69 (FN50, BD Pharmingen) after 16-18 h. For functional analysis of PBMCs, CD1d-endo tetramer positive or negative cells that had been FACS-sort expanded for 20 days as described above were exposed to 10 ng/mL PMA, 1 μg/mL Ionomicyn and 1/1000, Golgi stop (1/500, BD Pharmingen), Brefeldin A (1/1000, BD Pharmingen) and for 4 h in complete media prior to assessment of intracellular cytokine production using BD Cytofix/Cytoperm kit (BD Biosciences), anti-TNF (MAb11, BD Pharmingen), anti-IFNγ (4S.B3, Biolegend) and anti-IL-13 (JES10-5A2, BD Pharmingen).

### Isolation of monocytes and generation of MoDCs for activation assays

CD14^+^ were isolated from fresh PBMCs using magnetic enrichment in LS columns (Miltenyi) with anti-CD14 coated Microbeads (Miltenyi). To generate CD11c^+^ MoDCs, enriched cells were plated at 2 × 10^6^ cells per well in 24-well plates in 1.5 ml of in complete RPMI-1640 with GM-CSF (100 ng/ml) and IL-4 (50 ng/ml) (Biolegend). After 2 days, media was aspirated and replaced with fresh GM-CSF and IL-4 supplemented media. MoDCs were harvested at day 5 and assessed for CD1d and CD11c expression (anti-CD1d clone 51.1 Biolegend, anti CD11c clone B-ly6 BD). CD14^+^ cells or CD11c^+^ MoDCs were then co-cultured at a 1:1 ratio with T26A or NKT15, to test their ability to activate these cells, (CD69 upregulation after 16 h), in the presence or absence of α-GalCer C26:0 at 0.33 μg/mL, or vehicle.

### CD1d and NKT TCR production and purification

The human CD1d sequence encoding for truncated recombinant human CD1d ectodomain harbouring a C-terminal BirA and six-histidine (GSGLNDIFEAQKIEWHEHHHHHH) affinity tags, and β2-microglobulin, both in separate pHLsec vectors, were co-transfected into GnTI^−/−^ HEK293S for expression using polyethyleneimine as described^7,80^. The soluble CD1d glycoprotein was purified by Ni/NTA affinity purification followed by size-exclusion chromatography using a Superdex 200 16/60 column (GE Healthcare). CD1d was enzymatically biotinylated using biotin ligase (produced in-house) and further purified by size-exclusion chromatography using a Superdex-75 16/60 gel-filtration column (GE healthcare), followed by storage at −80 °C. Biotinylated CD1d proteins carrying endogenous lipids (endo), or exogenously loaded in vitro (as described in the lipids section) were tetramerised using SAV-PE or SAV-BV421 (Biolegend).

The genes encoding the T26A (TRAV26-TRBV19), T14 (TRAV14-TRBV19) and T26B (TRAV26-TRBV13) TCRs after codon optimisation, were synthesised (Genscript) and cloned into the expression vector pET30 (Novagen). The TCRα and TCRβ chains of the three NKT TCRs were expressed in *Escherichia coli* strain BL21 and purified as inclusion bodies (IBs) as described^17^. The IBs were resuspended in the following buffer: 8 M urea, 0 mM Tris-HCl-pH 8.0, 0.5 mM Na-EDTA, 1 mM DTT. The three TCRs were refolded by dilution in a solution containing 5 M urea, 0.1 M Tris-HCl-pH 8.0, 2 mM Na-EDTA, 400 mM L-arginine-HCl, 0.5 mM oxidised glutathione, 5 mM reduced glutathione. The refolding solutions were then dialysed against 10 mM Tris pH 8.0. The NKT TCRs were purified by Diethyl aminoethyl (DEAE) anion exchange, size exclusion and HiTrap-Q anion-exchange chromatography techniques.

### In vitro loading of lipids into human CD1d for crystallography and SPR

Lipid solutions of GD3, sulfatide and α-GalCer were prepared at 1 mg/mL in 0.5% tyloxapol. Prior to lipid loading, the lipid solutions were sonicated for 30 mins and immediately transferred to a 60 °C water bath for 1 min and left to cool at room temperature for 1 min. The lipids GD3, sulfatide were then added directly to CD1d-endo at a 3-6:1 molar ratio and incubated in 10 ml TBS150 (Tris-buffered saline, 150 mM NaCl, 10 mM Tris pH 8.0) at room temperature for 15 h. The lipid-loaded CD1d were then purified by MonoQ anion-exchange chromatography (GE Healthcare). α-GalCer was loaded at a 6:1 molar ratio into the purified CD1d-GD3 binary complex and purified by MonoQ anion-exchange chromatography.

### Purification of TCR-CD1d-endo complex and mass spectrometry analysis

The T26A, T26B or T14 TCRs were incubated with CD1d-endo for 16 h. The mixture was then subjected to gel filtration on a Superdex 200 16/60 column (GE Healthcare) to isolate the TCR-CD1d-endo ternary complex. The ‘TCR trap’ assay was performed as previously described with minor changes^42,43^. The gel filtration protein fractions were subjected to Bligh and Dyer extraction, and the extracted lipids were initially analysed by shotgun nano-electrospray on a Thermo Fisher LXQ Ion Trap mass spectrometer. For the semi-quantitative, high-resolution mass analysis, the lipid eluents were normalised to 20 µM based on the input proteins and 10 µl was injected into a reversed-phase HPLC system (Agilent Poroshell EC-C18 column, 1.9-micron, 3 × 50 mm) with an Agilent 6520 Accurate-Mass Q-TOF mass spectrometer. The gradient conditions were modified from a prior method (Klooster, elife, 2020): the mobile phases were (A) 2 mM ammonium formate in 90/10 methanol/water (v:v) and (B) 2 mM ammonium formate in 90/10/0.1 1-propanol/cyclohexane/water (v:v:v). The 30-minute gradients were: 0-4 min of 100% A, 4–13 min from 100% A to 100% B, 13–18 min of 100% B, 18–20 min from 100% B to 100% A, and 20–30 min of 100% A.

### Surface plasmon resonance (SPR) measurements and analysis

All the experiments were performed at 25 °C on Biacore 3000 and T200 using HBS buffer (10 mM HEPES- HCl - pH 7.4, 150 mM NaCl). Biotinylated CD1d-endo, CD1d-sulfatide, CD1d-α-GalCer and CD1d-GD3 were coupled (~3000 RU) onto a research-grade SA chip and the four TCRs were applied at a flow rate of 5 μl/min. The first flow cell was left blank and was used as a reference for subsequent analysis. The final response was calculated by subtraction of the response of the streptavidin-coated chip alone from that of TCR-CD1d-lipid interaction. BIAevaluation Version 3.1 (Biacore AB) and Biacore T200 Evaluation Software 3.2.1 were used to fit the data to the 1:1 Langmuir binding model. GraphPad Prism 11 was used for data presentation.

### Crystallisation, structure determination and refinement

The T26A NKT TCR-CD1d-endo (7 mg/ml) and the T26A NKT TCR-CD1d-GD3 (7 mg/ml) ternary complexes were both crystallised in 16-24% PEG 3350, 0.1 M sodium citrate, 0.1 M citrate-bis-Tris-pH 7 using the hanging-drop diffusion method at 20 °C. The crystals were cryoprotected in 10% glycerol and flash-frozen in liquid nitrogen. Crystallographic data were collected at the MX2 beamline (Australian Synchrotron). Data were processed with the XDS software^81^ and scaled using SCALA from the CCP4 suite of programmes^82^. The crystal structure of the T26A NKT TCR-CD1d-endo ternary complex was determined by molecular replacement method using PHASER^83^ and the TCRα (Protein Data Bank accession code: 6RSY)^84^, TCRβ (PDB accession code: 5JHD)^85^ and human CD1d–α-GalCer (PDB accession code: 2PO6)^35^ as search models. The crystal structure of the T26A NKT TCR-CD1d-GD3 ternary complex was also solved by molecular replacement using PHASER and the T26A NKT TCR-CD1d-endo ternary complex as a search model. Both ternary complexes were refined using the Phenix refinement programme^86^, and the COOT programme^87^ was used for macromolecular model building. The quality of the structures was validated using the Research Collaboratory for Structural Bioinformatics Protein Data Bank Data Validation and Deposition Services. All structural diagrams were created using PyMOL^88^.

### Reporting summary

Further information on research design is available in the Nature Portfolio Reporting Summary linked to this article.

## Supplementary information

Supplementary Information

Transparent Peer Review file

Reporting Summary

## Source data

Source Data

## Data Availability

The coordinates of the T26A NKT TCR-CD1d-SM and T26A NKT TCR-CD1d-GD3 ternary complexes were deposited in the Protein Data Bank (PDB) database under the accession codes 8SGB https://doi.org/10.2210/pdb8sgb/pdb and 8SGM https://doi.org/10.2210/pdb8SGM/pdb, respectively. Other raw data are available within the article and associated files and upon request from the corresponding authors. Source data are provided with this paper.
