# Supplementary material for: A public human population of TRAV26+ type II natural killer T cells recognises CD1d in a lipid-independent manner
Source: Nat Commun. 2026 Oct 7;17:11430. doi: 10.1038/s41467-026-78124-5 (PMC13736892; doi:10.1038/s41467-026-78124-5)
Supplement: Supplementary file 1 [file 41467_2026_78124_MOESM1_ESM.pdf]

Supplementary information

A public human population of TRAV26$^+$ type II natural killer T cells recognises CD1d in a lipid-independent manner

Kean Chan Yew Poa[1, *], Christopher M Harpur[2,3, *], Tan-Yun Cheng[4], Elena Batleska[2,3], Catriona V Nguyen-Robertson[2], Rajesh Lamichhane[2], Alison A White[2], Geovane Marques-Ferreira[2,5], Caroline Soliman[2,ψ], Scott JJ Reddiex[2], Christopher Menne[3], Adam P Uldrich[2], D Branch Moody[4], Jamie Rossjohn[1], Dale I Godfrey[2], Daniel G Pellicci[2, 3,6, #,@], Jérôme Le Nours[1, #,@], Catarina F Almeida[2, #,@]

[1]Infection and Immunity Program and Department of Biochemistry and Molecular Biology, Biomedicine Discovery Institute, Monash University, Clayton, Victoria 3800, Australia.

[2]Department of Microbiology & Immunology, Peter Doherty Institute for Infection and Immunity, The University of Melbourne, Melbourne, Victoria 3010, Australia.

[3]Infection, Immunity and Global Health, Murdoch Children's Research Institute, Parkville, Victoria 3052, Australia.

[4]Division of Rheumatology, Inflammation, and Immunity, Brigham and Women's Hospital and Harvard Medical School, Boston, MA 02115, United States.

[5]Departamento de Microbiologia, Instituto de Ciências Biológicas, Universidade Federal de Minas Gerais, Avenida Antônio Carlos, 6627 – Pampulha, Belo Horizonte, Minas Gerais 31270-901, Brazil

[6]Department of Paediatrics, The University of Melbourne, Melbourne, Victoria 3010, Australia.

[ψ]Present address: CSL Innovation Pty Ltd., Melbourne, Victoria 3000, Australia

[*]These authors contributed equally

[#] These authors jointly supervised this work

[@]Corresponding authors emails: catarina.dos@unimelb.edu.au, jerome.lenours@monash.edu, dan.pellicci@mcri.edu.au

Supplementary information

A public human population of TRAV26^{+} type II natural killer T cells recognises CD1d in a lipid-independent manner

Supplementary file- table of contents

4 Supplementary Figures

Figure S1 Gating Strategies

Figure S2. Electron density maps of CD1d-ligand, CD1d-GD3-loading validation, T26A-CD1d-GD3 interactions, CD1d-ligand overlays

Figure S3. CD1d-dependent activation of CD1d-endo-reactive Type 2 NKT cells is not lipid sensitive

Figure S4. Primary MoDCs upregulate CD1d and activate T26 cells in the absence of exogenous ligands

5 Supplementary Tables

Table S1 Data collection and refinement statistics.

Table S2 T26A NKT TCR molecular contacts with and CD1d-SM.

Table S3 T26A NKT TCR molecular contacts with and CD1d-GD3.

Table S4. Antibodies used in the study

Table S5. Primers used in the study

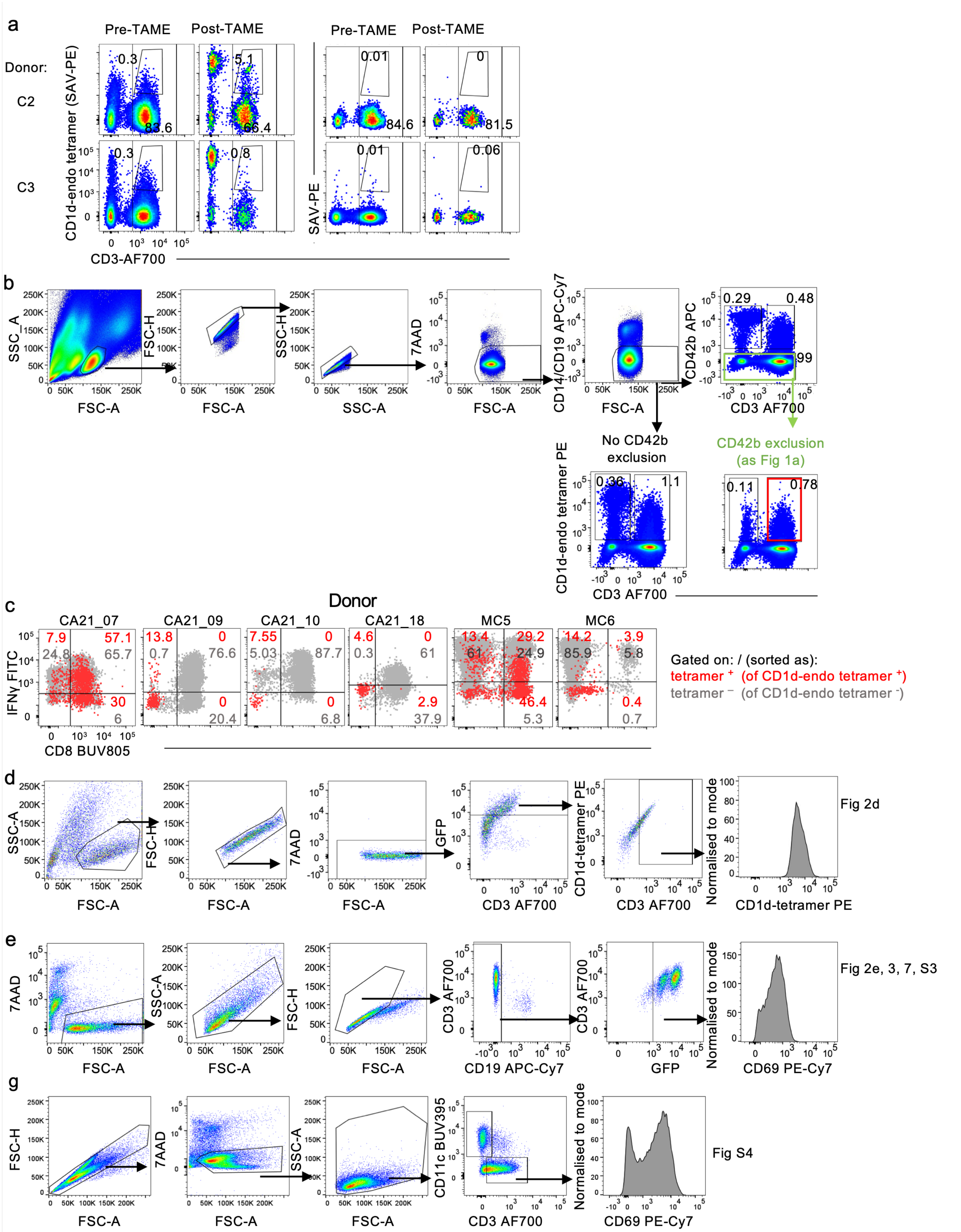

Fig. S1. Gating Strategies (a) Flow cytometry plots of three donors showing CD3 *vs* CD1d-endo tetramer and streptavidin (SAV)-PE control staining pre- and post-CD1d-endo tetramer associated magnetic enrichment (TAME), gated on 7AAD⁻CD14⁻CD19⁻ single lymphocytes. Data shown is from one experiment, representative of 5 donors acquired over 2 experiments where SAV-PE enrichments were performed alongside CD1d-endo TAME. **(b)** Gating strategy for flow cytometry analysis of CD1d-endo tetramer staining of PBMCs with and without CD42b exclusion

gate applied. Data is from donor CH7 and representative of the data graphed in Figures 1 and 2c. **(c)** Overlayed dot plots show CD8 *versus* IFNγ on stimulated CD1d-endo tetramer $^{+}$ cells (in red, gated as per the red gate in (1d)), stimulated (grey) or unstimulated (black) CD1d-endo tetramer $^{-}$ sorted cells. Data shown is from 6 donors, analysed across 4 experiments. (**d**) Gating strategy for flow cytometry analysis of CD1d-tetramer stain TCR reporter lines HEK293T or SKW3.β2m $^{-/-}$. The plot shown is from T26A stained with CD1d-endo tetramer and representative of data in figure 2d. (**e**) Gating strategy for flow cytometry analysis of TCR reporter lines HEK293T or SKW3.β2m $^{-/-}$ co-cultures with C1R lines. The plot shown is from NKT15:C1R.wt co-culture in the presence of SM and is representative of a datapoint in Figure 2e. **(g)** Gating strategy for flow cytometry analysis of TCR reporter lines SKW3.β2m $^{-/-}$ co-cultures with $CD14^{+}$ monocytes (Mono) and MoDCs. The plot shown is from NKT15:$CD14^{+}$ Mono with α-GalCer.

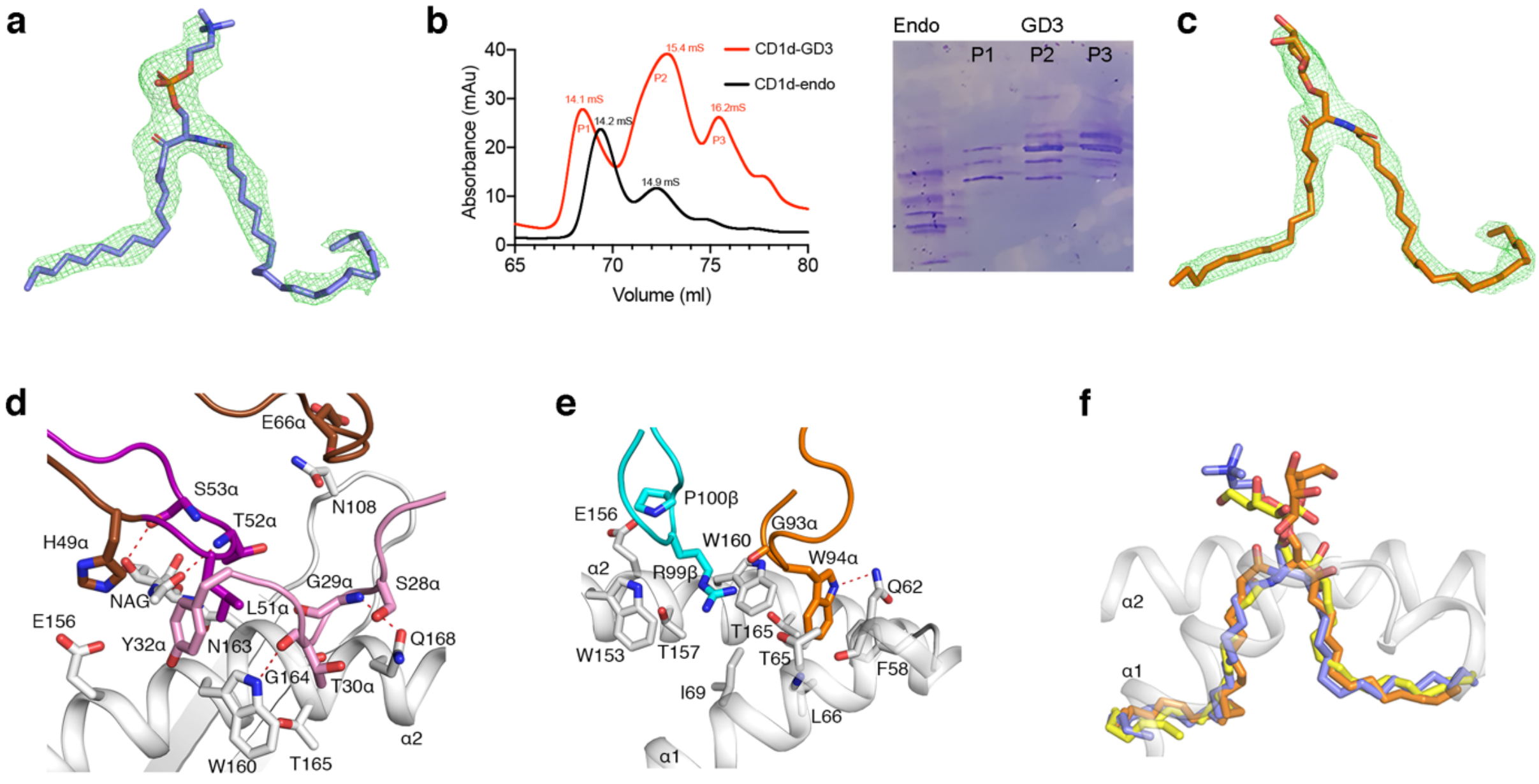

Fig. S2. Electron density maps of CD1d-ligand, CD1d-GD3-loading validation, T26A-CD1d-GD3 interactions, CD1d-ligand overlays (a) Fo-Fc electron density map of SM contoured at 2.2σ level. **(b)** Anion exchange chromatography profiles of CD1d-endo and CD1d-GD3 and the isoelectric focussing (IEF) gel of CD1d-endo with peaks P1, P2 and P3 of CD1d-GD3 isolated from the anion exchange purification. **(c)** Fo-Fc electron density map of GD3 (partially modelled) contoured at 2.2σ level. **(d-e)** Molecular interactions of the T26A CDR1α (pink), CDR2α (purple), CDR3α (orange), CDR3β (cyan) and FWα (brown) with CD1d-GD3. **(f)** Superposition of the CD1d-SM (in light blue, PDB code:8SGB), the partially modelled CD1d-GD3 (in orange, PDB code: 8SGM) and α-GalCer (in yellow, PDB code: 1ZT4).

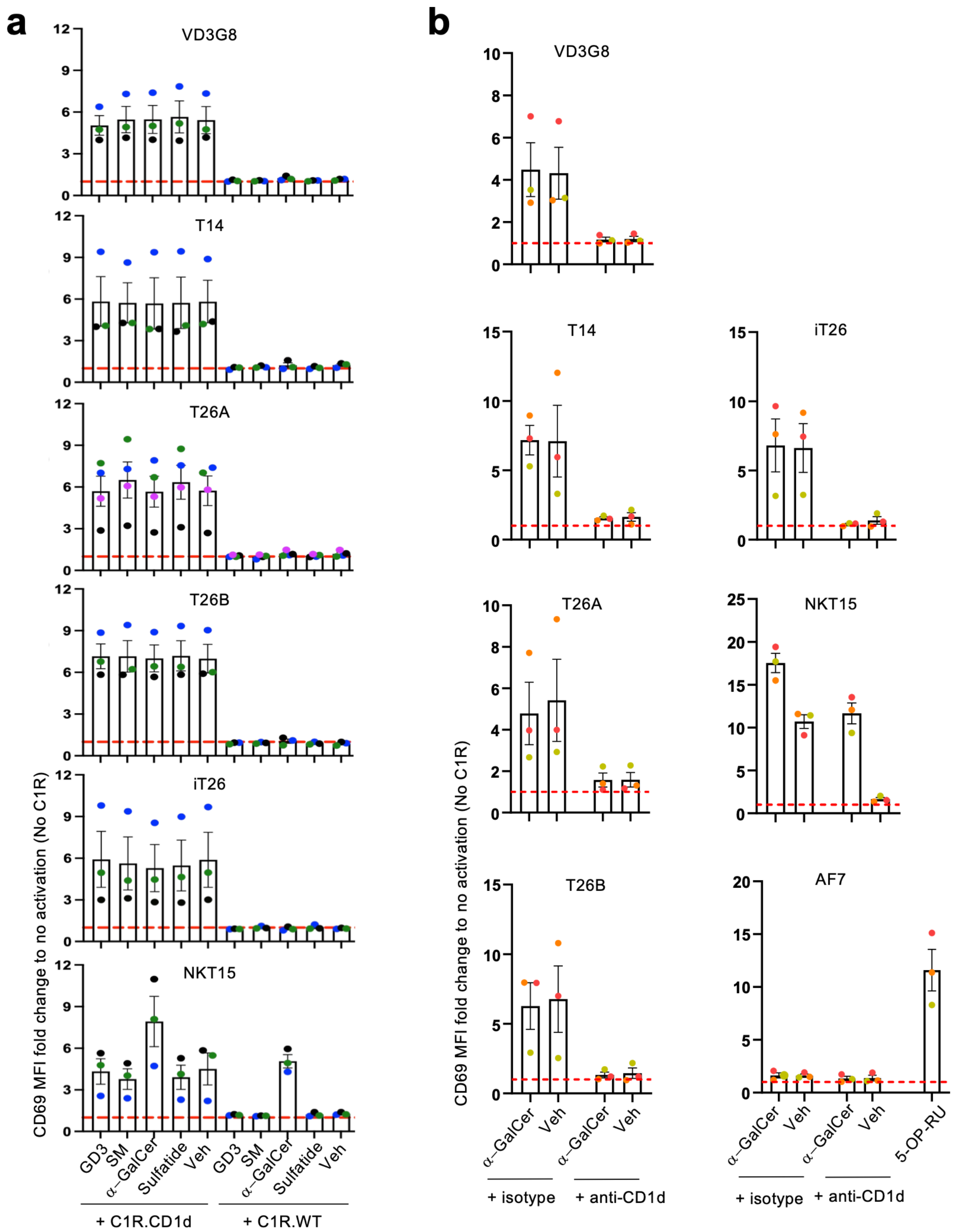

Fig. S3. CD1d-dependent activation of CD1d-endo-reactive Type 2 NKT cells is not lipid sensitive (a) SKW3.β2m$^{-/-}$ TCR-transduced cell lines were co-cultured with wild type C1R cells (C1R.WT) or C1R transduced to express high levels of CD1d (C1R.CD1d) in the presence or absence of GD3, sphingomyelin (SM), sulfatide (all at 20 µg/mL), α-GalCer (1 µg/mL) or vehicle control (Veh). Graphs show fold increase in CD69 mean fluorescence intensity (MFI) relative to co-cultures with C1R.WT in the absence of exogenous Ag+/- SEM. Red line depicts fold-increase = 1 (baseline). Data shown is from $n = 3$ independent experiments, $n = 4$ for T26A, each represented by a different coloured dot. **(b)** SKW3.β2m$^{-/-}$ TCR-transduced cell lines were co-cultured with C1R.WT or C1R.CD1d in the presence or absence of α-GalCer (1µg/mL), or vehicle control (Veh) together with either anti-CD1d (25µg/mL) or isotype control (isotype). The AF7 MAIT SKW3.β2m$^{-/-}$ TCR-transduced cell line and 5-OP-RU (1nM) were also included as controls. Graphs show fold increase in CD69 MFI relative to SKW3.β2m$^{-/-}$ TCR-transduced cell lines in the absence of C1R.CD1d cells, exogenous antigen or antibody +/- SEM. Red line depicts fold-increase = 1 (baseline). Data shown is from $n = 3$ independent experiments, each represented by a different coloured dot. The conditions pertaining co-cultures with α-GalCer and no lipid are also shown in Fig. 2e retaining the same colour-coding.

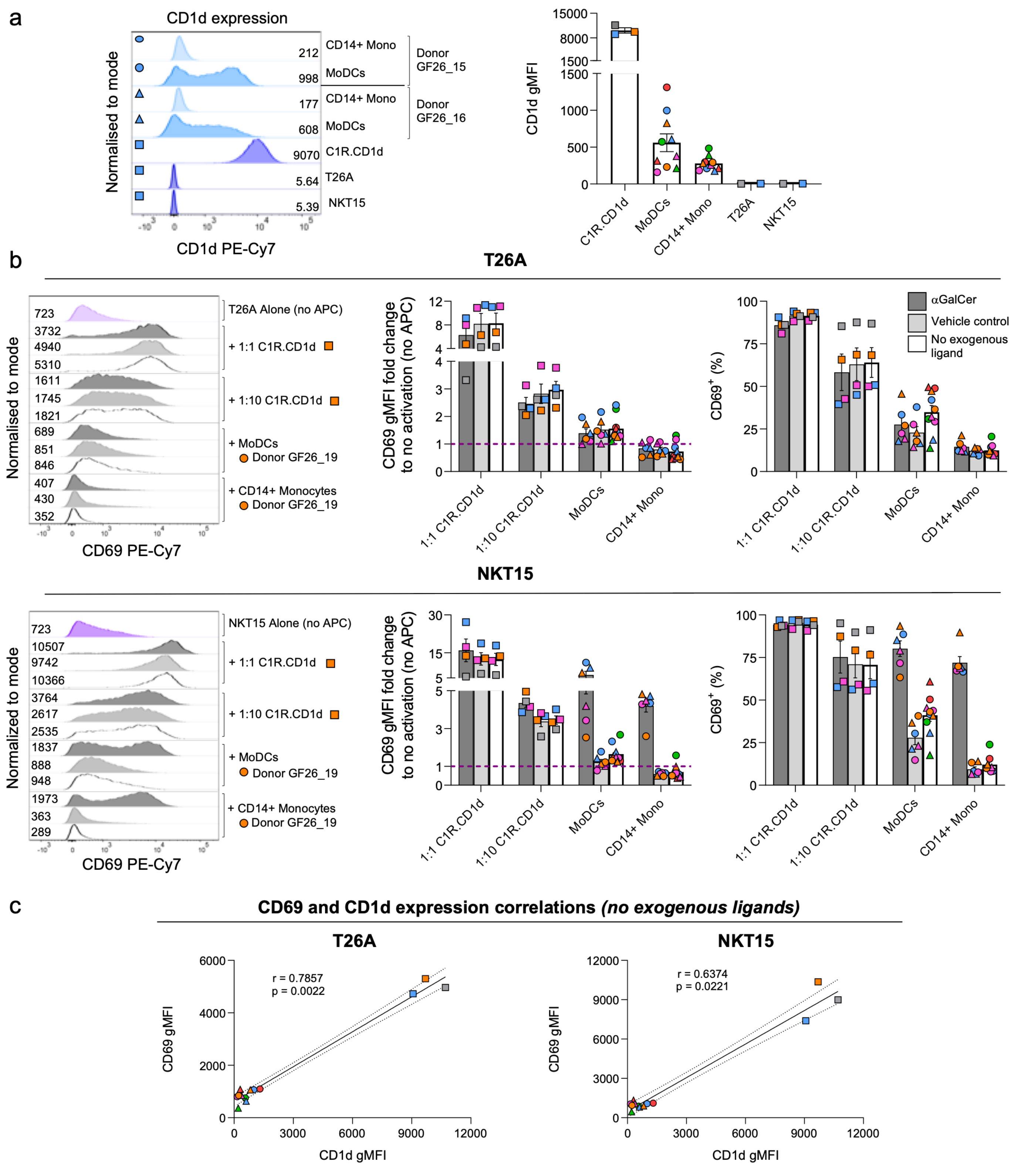

Figure S4 Primary MoDCs upregulate CD1d and activate T26 cells in the absence of exogenous ligands (a) Surface expression of CD1d was assessed on magnetically enriched CD14$^+$ monocytes, as well as monocyte-derived dendritic cells (MoDCs, day 5 post differentiation with GM-CSF/IL-4), CD1d-transduced C1R cells (C1R.CD1d), or T26A and NKT15 (SKW3.β2m$^{-/-}$). Histograms show CD1d geometric mean fluorescence intensity (gMFI, indicated by the numbers), from one representative experiment including two donor-derived CD14$^+$ Mono or MoDCs. Graphs show CD1d gMFI ± SEM from *n=5* experiments with CD14$^+$ Mono or MoDCs, each including two donors per experiment (represented by different shapes, 10 donors in total), *n=3* experiments for C1R.CD1d and *n=2* for each SKW3.β2m$^{-/-}$ NKT TCR reporter line. Conditions analysed alongside each experiment are matched by colour. **(b)** T26A or NKT15 were co-cultured with C1R.CD1d cells (at 1:1 or 1:10 antigen presenting cell (APC):effector ratios), and separately with donor-derived MoDCs or CD14$^+$monocytes (1:1 ratio), in the presence or absence of α-Galactosylceramide (α-GalCer; 0.33μg/mL), or vehicle control. Overlayed histograms show CD69 gMFI (indicated

by numbers) from one representative experiment. Vertical dashed line represents gate placement for CD69 % determination. Graphs show CD69 gMFI fold increase relative to each SKW3.β2m$^{-/-}$ NKT reporter line alone (horizontal dashed purple line), or CD69 %, ± SEM. Data is from *n=4* experiments for all C1R:CD1d co-culture conditions, and *n=5* for CD14^{+} Mono or MoDCs in the absence of antigen, *n=3* in the presence of α-GalCer or Vehicle. Data is shape and colour coded as per (**a**). **(c)** Spearman correlation analyses were performed between CD1d surface expression (CD1d gMFI) on APCs, including CD14^{+} Mono, MoDCs or C1R.CD1d cells in 1:1 co-cultures in the absence of exogenous antigen, and the level of activation of each NKT TCR reporter lines (CD69 gMFI). Dotted lines represent simple linear regression with 95% confidence intervals. Data is derived from *n=3* experiments for C1R.CD1d and *n=5* for MoDCs (including 10 donors). Data is shape and colour coded as per (**a**).

Table S1. Data collection and refinement statistics.

	T26A NKT TCR-CD1d-SM	T26A NKT TCR-CD1d-GD3
Data collection		
Temperature	100K	100K
Resolution limits (Å)	38.2-2.8 (2.9-2.8)	45.1-2.5 (2.6-2.5)
Space Group	$P4_1$	$P4_1$
Cell dimensions (Å)	*a*=136.79, *b*=136.79, *c*=69.80	*a*=133.79, *b*=133.79, *c*=68.57
Total N$^{o.}$ observations	443095 (56686)	298286 (31367)
N$^{o.}$ unique observations	32086 (4639)	42283 (4426)
Multiplicity	13.8 (12.2)	7.1 (7.1)
Data completeness	100 (100)	100 (100)
Wilson B-factors (Å^2)	66.5	49.2
I/σ_I	7.0 (2.3)	11.5 (3.70)
$R_{p.i.m}$ [1] (%)	6.4 (25.7)	3.9 (18.7)
Refinement statistics		
R_{factor} [2] (%)	21.7	18.6
R_{free} [3] (%)	25.6	23.3
Non hydrogen atoms		
- Protein	6296	6306
- Water	32	413
- Heterogen	124	152
Ramachandran plot (%)		
- Most favoured	91	95
- Allowed	7.8	4.8
B-factors (Å^2)		
- protein	67.7	51.4
- ligands	77	64.3
rmsd bonds (Å)	0.004	0.008
rmsd angles (°)	0.76	0.99

[1] $R_{p.i.m} = \Sigma_{hkl} [1/(N-1)]^{1/2} \Sigma_i | I_{hkl, i} - \langle I_{hkl} \rangle | / \Sigma_{hkl} \langle I_{hkl} \rangle$.

[2] $R_{factor} = (\Sigma \,|\,|F_o| - |F_c|\,|) / (\Sigma |F_o|)$ - for all data except as indicated in footnote 3.

[3] 5% of data was used for the R_{free} calculation.

Values in parentheses refer to the highest resolution bin.

Table S2. T26A NKT TCR molecular contacts with and CD1d-SM.

TCR gene	TCR residues	CD1d residues	Bond type
CDR1α	Ser^{28}	Gln^{168}	VDW
CDR1α	Gly^{29}	Gly^{164}, Gln^{168}	VDW
CDR1α	$Gly^{29\text{-}N}$	$Gln^{168\text{-}O\varepsilon 1}$	HB
CDR1α	Thr^{30}	Trp^{160}, Gly^{164}, Thr^{165}, Gln^{168}	VDW
CDR1α	Tyr^{32}	Glu^{156}, Trp^{160}	VDW
FWα	$His^{49\text{-}N\varepsilon 2}$	$Glu^{156\text{-}O\varepsilon 2}$	HB
CDR2α	Leu^{51}	Asn^{163}, NAG^{278}	VDW
CDR2α	Thr^{52}	Asn^{163}, NAG^{278}	VDW
CDR2α	$Thr^{52\text{-}N}$	$NAG^{278\text{-}O}$	HB
CDR2α	Ser^{53}	NAG^{278}	VDW
CDR2α	$Ser^{53\text{-}O\gamma}$	$NAG^{278\text{-}O}$	HB
CDR3α	Gly^{93}	Trp^{160}	VDW
CDR3α	Trp^{94}	Gln^{62}, Leu^{66}, Trp^{160}	VDW
CDR3β	Arg^{99}	Ile^{69}, Trp^{153}, Thr^{157}, Trp^{160}	VDW
CDR3β	$Arg^{99\text{-}N\eta 1}$	$Thr^{157\text{-}O\gamma 1}$	HB
CDR3β	Pro^{100}	Glu^{156}	VDW

HB: Hydrogen bond, VDW: van der Waals,

Cut-off at 4 Å for VDW interactions and 3.5 Å for HB, NAG: N-acetyl-glucosamine.

Table S3. T26A NKT TCR molecular contacts with and CD1d-GD3.

TCR gene	TCR residues	CD1d residues	Bond type
CDR1α	Ser^{28}	Gln^{168}	VDW
CDR1α	Gly^{29}	Gly^{164}, Gln^{168}	VDW
CDR1α	$Gly^{29\text{-N}}$	$Gln^{168\text{-O}\varepsilon 1}$	HB
CDR1α	Thr^{30}	Trp^{160}, Gly^{164}, Thr^{165}	VDW
CDR1α	$Thr^{30\text{-O}}$	$Trp^{160\text{-N}\varepsilon 1}$	HB
CDR1α	Tyr^{32}	Glu^{156}, Trp^{160}	VDW
FWα	His^{49}	Glu^{156}	VDW
CDR2α	Leu^{51}	Trp^{160}, Asn^{163}, NAG^{278}	VDW
CDR2α	Thr^{52}	Asn^{163}, NAG^{278}	VDW
CDR2α	$Thr^{52\text{-N}}$	$NAG^{278\text{-O}}$	HB
CDR2α	Ser^{53}	NAG^{278}	VDW
CDR2α	$Ser^{53\text{-O}\gamma}$	$NAG^{278\text{-O}}$	HB
FWα	Glu^{66}	Asn^{108}	VDW
CDR3α	Gly^{93}	Trp^{160}	VDW
CDR3α	Trp^{94}	Phe^{58}, Gln^{62}, Thr^{65}, Leu^{66}, Trp^{160}, Thr^{165}	VDW
CDR3α	$Trp^{94\text{-N}\varepsilon 1}$	$Gln^{62\text{-N}\varepsilon 2}$	HB
CDR3β	Arg^{99}	Ile^{69}, Trp^{153}, Thr^{157}, Trp^{160}	VDW
CDR3β	Pro^{100}	Glu^{156}	VDW

HB: Hydrogen bond, VDW: van der Waals,

Cut-off at 4 Å for VDW interactions and 3.5 Å for HB, NAG: N-acetyl-glucosamine.

Table S4. Antibodies used in the study

Target	Clone	Fluorochrome	Dilution	Manufacturer	Catalogue #
CD1d	51.1	PE-Cy7	1:200	BioLegend	350310
		Purified	Final 25µg/mL	BioLegend	350322
CD11c	B-ly6	BUV395	1:200	BD Horizon	563787
CD3	UCHT1	AF700	1:100	BD Pharmingen	557943
		BUV395	1:100	BD Horizon	563546
		Purified	Final 10µg/mL	BD Pharmingen	550368
	OKT3	Purified	Final 4µg/mL	BioXCell	BE0001-2
CD28	CD28.2	Purified	Final 2µg/mL	BD Pharmingen	555726
	9.3	Purified	Final 4µg/mL	BioXCell	BE0248
CD4	SK3	BUV496	1:100	BD Horizon	612936
CD8α	SK1	BUV805	1:200	BD Horizon	612889
CD14	MφP9	APC-Cy7	1:100	BD Pharmingen	557831
	M5E2	BV570	1:200	BioLegend	301832
CD19	SJ25C1	APC-Cy7	1:100	BD Pharmingen	557791
CD36	5-271	Unconjugated	1:100	BioLegend	336202
CD42b	HIP1	APC	1:50	BD Pharmingen	551061
CD69	FN50	PE-Cy7	1:100	BD Horizon	557745
Fc Receptor Block			1:25-1:50	Miltenyi Biotec	130-059-901
IFNγ	4S.B3	FITC	1:100	BioLegend	502506
IL-13	JES10-5A2	BV711	1:100	BD Horizon	564288
PLZF	R17-809	PEcf594	1:4000	BD Horizon	565738
TCRγδ	11F2	FITC	1:50	BD	347903
	11F2	PE-Cy7	1:50	BD	655410
TNF	MAb11	AF700	1:200	BD Pharmingen	557996
IgG2B	MPC-11	Purified	Final 25µg/mL	BioLegend	400326
	MG2b-57	Purified	Final 25µg/mL	BioLegend	401217

Table S5. Primers used in the study.

TCRα and TCRβ primers are from Wang et al Sci Transl Med 2012, DOI: 10.1126/scitranslmed.3003647, TCRγ and TCRδ primers from Guo et al Molecular Therapy — Methods & Clinical Development (2016) 3, 15054; doi:10.1038/mtm.2015.54

<table>
<tr><th colspan="2">TCR Variable α (TRAV) Forward primer</th></tr>
<tr><th>Internal(Int)/External (Ext)</th><th>Sequence</th></tr>
<tr><td>hTRAV1_Ext</td><td>AACTGCACGTACCAGACATC</td></tr>
<tr><td>hTRAV1_Int</td><td>GCACCCACATTTCT/|K|/TCTTAC</td></tr>
<tr><td>hTRAV2_Ext</td><td>GATGTGCACCAAGACTCC</td></tr>
<tr><td>hTRAV2_Int</td><td>CACTCTGTGTCCAATGCTTAC</td></tr>
<tr><td>hTRAV3_Ext</td><td>AAGATCAGGTCAACGTTGC</td></tr>
<tr><td>hTRAV3_Int</td><td>ATGCACCTATTCAGTCTCTGG</td></tr>
<tr><td>hTRAV4_Ext</td><td>CTCCATGGACTCATATGAAGG</td></tr>
<tr><td>hTRAV4_Int</td><td>ATTATATCACGTGGTACCAACAG</td></tr>
<tr><td>hTRAV5_Ext</td><td>CTTTTCCTGAGTGTCCGAG</td></tr>
<tr><td>hTRAV5_Int</td><td>TACACAGACAGCTCCTCCAC</td></tr>
<tr><td>hTRAV6_Ext</td><td>CACCCTGACCTGCAACTATAC</td></tr>
<tr><td>hTRAV6_Int</td><td>TGGTACCGACAAGATCCAG</td></tr>
<tr><td>hTRAV7_Ext</td><td>AGCTGCACGTACTCTGTCAG</td></tr>
<tr><td>hTRAV7_Int</td><td>ACAATTTGCAGTGGTACAGG</td></tr>
<tr><td>hTRAV8-1_Ext</td><td>CTCACTGGAGTTGGGATG</td></tr>
<tr><td>hTRAV8-1_Int</td><td>GTCAACACCTTCAGCTTCTC</td></tr>
<tr><td>hTRAV8-2_8-4_Ext</td><td>GCCACCCTGGTTAAAGG</td></tr>
<tr><td>hTRAV8-2_8-4_Int</td><td>AGAGTGAAACCTCCTTCCAC</td></tr>
<tr><td>hTRAV8-3_Ext</td><td>CACTGTCTCTGAAGGAGCC</td></tr>
<tr><td>hTRAV8-3_Int</td><td>TTTGAGGCTGAATTTAAGAGG</td></tr>
<tr><td>hTRAV8-6_Ext</td><td>GAGCTGAGGTGCAACTACTC</td></tr>
<tr><td>hTRAV8-6_Int</td><td>AACCAAGGACTCCAGCTTC</td></tr>
<tr><td>hTRAV8-7_Ext</td><td>CTAACAGAGGCCACCCAG</td></tr>
<tr><td>hTRAV8-7_Int</td><td>ATCAGAGGTTTTGAGGCTG</td></tr>
<tr><td>hTRAV9-1_9-2_ext</td><td>TGGTATGTCCAATATCCTGG</td></tr>
<tr><td>hTRAV9-1_9-2_Int</td><td>GAAACCACTTCTTTCCACTTG</td></tr>
<tr><td>hTRAV10_Ext</td><td>CAAGTGGAGCAGAGTCCTC</td></tr>
<tr><td>hTRAV10_Int</td><td>GAAAGAACTGCACTCTTCAATG</td></tr>
<tr><td>hTRAV12-1_12-2_12-3_</td><td>CA/|R|/TGTTCCAGAGGGAGC</td></tr>
<tr><td>hTRAV12-1_12-2_12-3_</td><td>AAGATGGAAGGTTTACAGCAC</td></tr>
<tr><td>hTRAV13-1_Ext</td><td>CATCCTTCAACCCTGAGTG</td></tr>
<tr><td>hTRAV13-1_Int</td><td>TCAGACAGTGCCTCAAACTAC</td></tr>
<tr><td>hTRAV13-2_Ext</td><td>CAGCGCCTCAGACTACTTC</td></tr>
<tr><td>hTRAV13-2_Int</td><td>CAGTGAAACATCTCTCTCTGC</td></tr>
<tr><td>hTRAV14_Ext</td><td>AAGATAACTCAAACCCAACCAG</td></tr>
<tr><td>hTRAV14_Int</td><td>AGGCTGTGACTCTGGACTG</td></tr>
<tr><td>hTRAV16_Ext</td><td>AGTGGAGCTGAAGTGCAAC</td></tr>
<tr><td>hTRAV16_Int</td><td>GTCCAGTACTCCAGACAACG</td></tr>
<tr><td>hTRAV17_Ext</td><td>GGAGAAGAGGATCCTCAGG</td></tr>
<tr><td>hTRAV17_Int</td><td>CCACCATGAACTGCAGTTAC</td></tr>
<tr><td>hTRAV18_Ext</td><td>TCCAGTATCTAAACAAAGAGCC</td></tr>
<tr><td>hTRAV18_Int</td><td>TGACAGTTCCTTCCACCTG</td></tr>
<tr><td>hTRAV19_Ext</td><td>AGGTAACTCAAGCGCAGAC</td></tr>
<tr><td>hTRAV19_Int</td><td>TGTGACCTTGGACTGTGTG</td></tr>
<tr><td>hTRAV20_Ext</td><td>CACAGTCAGCGGTTTAAGAG</td></tr>
<tr><td>hTRAV20_Int</td><td>TCTGGTATAGGCAAGATCCTG</td></tr>
<tr><td>hTRAV21_Ext</td><td>TTCCTGCAGCTCTGAGTG</td></tr>
<tr><td>hTRAV21_Int</td><td>AACTTGGTTCTCAACTGCAG</td></tr>
<tr><td>hTRAV22_Ext</td><td>GTCCTCCAGACCTGATTCTC</td></tr>
<tr><td>hTRAV22_Int</td><td>CTGACTCTGTGAACAATTTGC</td></tr>
<tr><td>hTRAV23_Ext</td><td>TGCTTATGAGAACACTGCG</td></tr>
<tr><td>hTRAV23_Int</td><td>TGCATTATTGATAGCCATACG</td></tr>
<tr><td>hTRAV24_Ext</td><td>CTCAGTCACTGCATGTTCAG</td></tr>
<tr><td>hTRAV24_Int</td><td>TGCCTTACACTGGTACAGATG</td></tr>
<tr><td>hTRAV25_Ext</td><td>GGACTTCACCACGTACTGC</td></tr>
<tr><td>hTRAV25_Int</td><td>TATAAGCAAAGGCCTGGTG</td></tr>
<tr><td>hTRAV26-1_Ext</td><td>GCAAACCTGCCTTGTAATC</td></tr>
<tr><td>hTRAV26-1_Int</td><td>CGACAGATTCACTCCCAG</td></tr>
<tr><td>hTRAV26-2_Ext</td><td>AGCCAAATTCAATGGAGAG</td></tr>
<tr><td>hTRAV26-2_Int</td><td>TTCACTTGCCTTGTAACCAC</td></tr>
<tr><td>hTRAV27_Ext</td><td>TCAGTTTCTAAGCATCCAAGAG</td></tr>
<tr><td>hTRAV27_Int</td><td>CTCACTGTGTACTGCAACTCC</td></tr>
<tr><td>hTRAV29_Ext</td><td>GCAAGTTAAGCAAAATTCACC</td></tr>
<tr><td>hTRAV29_Int</td><td>CTGCTGAAGGTCCTACATTC</td></tr>
<tr><td>hTRAV30_Ext</td><td>CAACAACCAGTGCAGAGTC</td></tr>
<tr><td>hTRAV30_Int</td><td>AGAAGCATGGTGAAGCAC</td></tr>
<tr><td>hTRAV34_Ext</td><td>AGAACTGGAGCAGAGTCCTC</td></tr>
<tr><td>hTRAV34_Int</td><td>ATCTCACCATAAACTGCACG</td></tr>
<tr><td>hTRAV35_Ext</td><td>GGTCAACAGCTGAATCAGAG</td></tr>
<tr><td>hTRAV35_Int</td><td>ACCTGGCTATGGTACAAGC</td></tr>
<tr><td>hTRAV36_Ext</td><td>GAAGACAAGGTGGTACAAAGC</td></tr>
<tr><td>hTRAV36_Int</td><td>ATCTCTGGTTGTCCACGAG</td></tr>
<tr><td>hTRAV38-1_38-2_Ext</td><td>GCACATATGACACCAGTGAG</td></tr>
<tr><td>hTRAV38-1_38-2_Int</td><td>CAGCAGGCAGATGATTCTC</td></tr>
<tr><td>hTRAV39_Ext</td><td>CTGTTCCTGAGCATGCAG</td></tr>
<tr><td>hTRAV39_Int</td><td>TCAACCACTTCAGACAGACTG</td></tr>
<tr><td>hTRAV40_Ext</td><td>GCATCTGTGACTATGAACTGC</td></tr>
<tr><td>hTRAV40_Int</td><td>GGAGGCGGAAATATTAAAGAC</td></tr>
<tr><td>hTRAV41_Ext</td><td>AATGAAGTGGAGCAGAGTCC</td></tr>
<tr><td>hTRAV41_Int</td><td>TTGTTTATGCTGAGCTCAGG</td></tr>
</table>

<table>
<tr><th colspan="2">TCR Variable β (TRBV) Forward primer</th></tr>
<tr><th>Internal(Int)/External (Ext)</th><th>Sequence</th></tr>
<tr><td>hTRBV2_Ext</td><td>TCGATGATCAATTCTCAGTTG</td></tr>
<tr><td>hTRBV2_Int</td><td>TTCACTCTGAAGATCCGGTC</td></tr>
<tr><td>hTRBV3-1_Ext</td><td>CAAAATACCTGGTCACACAG</td></tr>
<tr><td>hTRBV3-1_Int</td><td>AATCTTCACATCAATTCCCTG</td></tr>
<tr><td>hTRBV4-1-3_Ext</td><td>TCGCTTCTCACCTGAATG</td></tr>
<tr><td>hTRBV4-1-3_Int</td><td>CCTGCAGCCAGAAGACTC</td></tr>
<tr><td>hTRBV5-1_5-3-4_Ext</td><td>GATTCTCAGG/|K|/C/|K|/CCAGTTC</td></tr>
<tr><td>hTRBV5-1_5-3-4_Int</td><td>CTTGGAGCTGG/|R|//|S|/GACTC</td></tr>
<tr><td>hTRBV5-5-8_Ext</td><td>GTACCAACAGG/|Y|/CCTGGGT</td></tr>
<tr><td>hTRBV5-5-8_Int</td><td>TCTGAGCTGAATGTGAACG</td></tr>
<tr><td>hTRBV6-1-3_6-5-9_F13</td><td>ACTCAGACCCCAAAATTCC</td></tr>
<tr><td>hTRBV6-1-3_6-5-9_Int</td><td>GTGT/|R|/CCCAGGATATGAACC</td></tr>
<tr><td>hTRBV6-4_Ext</td><td>ACTGGCAAAGGAGAAGTCC</td></tr>
<tr><td>hTRBV6-4_Int</td><td>TGGTTATAGTGTCTCCAGAGC</td></tr>
<tr><td>hTRBV7-1-3_Ext</td><td>T/|R|/TGATCCAATTTCAGGTCA</td></tr>
<tr><td>hTRBV7-1-3_Int</td><td>TC/|Y|/ACTCTGA/|M|/G/|W|/TCCAGCG</td></tr>
<tr><td>hTRBV7-4_7-6-9_Ext</td><td>G/|S|//|W|/TCT/|Y|/TGCAGA/|R|/AGGCC</td></tr>
<tr><td>hTRBV7-4_7-6-9_Int</td><td>TG/|R|//|M|/GAT/|Y|/CAGCGCACA</td></tr>
<tr><td>hTRBV9_Ex</td><td>GATCACAGCAACTGGACAG</td></tr>
<tr><td>hTRBV9_Int</td><td>GTACCAACAGAGCCTGGAC</td></tr>
<tr><td>hTRBV10-1-3_Ext</td><td>TGT/|W|/CTGGTATCGACAAGACC</td></tr>
<tr><td>hTRBV10-1-3_Int</td><td>TCC/|Y|/CCTCACTCTGGAGTC</td></tr>
<tr><td>hTRBV11-1-3_Ext</td><td>CGATTTTCTGCAGAGACGC</td></tr>
<tr><td>hTRBV11-1-3_Int</td><td>GACTCCACTCTCAAGATCCA</td></tr>
<tr><td>hTRBV12-3-5_Ext</td><td>A/|R|/GTGACAGA/|R|/ATGGGACAA</td></tr>
<tr><td>hTRBV12-3-5_Int</td><td>C/|Y|/ACTCTGA/|R|/GATCCAGCC</td></tr>
<tr><td>hTRBV13_Ext</td><td>AGCGATAAAGGAAGCATCC</td></tr>
<tr><td>hTRBV13_Int</td><td>CATTCTGAACTGAACATGAGC</td></tr>
<tr><td>hTRBV14_Ext</td><td>CCAACAATCGATTCTTAGCTG</td></tr>
<tr><td>hTRBV14_Int</td><td>ATTCTACTCTGAAGGTGCAGC</td></tr>
<tr><td>hTRBV15_Ext</td><td>AGTGACCCTGAGTTGTTCTC</td></tr>
<tr><td>hTRBV15_Int</td><td>ATAACTTCCAATCCAGGAGG</td></tr>
<tr><td>hTRBV16_Ext</td><td>GTCTTTGATGAAACAGGTATGC</td></tr>
<tr><td>hTRBV16_Int</td><td>GAAAGATTTTCAGCTAAGTGCC</td></tr>
<tr><td>hTRBV17_Ext</td><td>CAGACCCCCAGACACAAG</td></tr>
<tr><td>hTRBV17_Int</td><td>TGTTCACTGGTACCGACAG</td></tr>
<tr><td>hTRBV18_Ext</td><td>CATAGATGAGTCAGGAATGCC</td></tr>
<tr><td>hTRBV18_Int</td><td>CGATTTTCTGCTGAATTTCC</td></tr>
<tr><td>hTRBV19_Ext</td><td>AGTTGTGAACAGAATTTGAACC</td></tr>
<tr><td>hTRBV19_Int</td><td>TTCCTCTCACTGTGACATCG</td></tr>
<tr><td>hTRBV20-1_Ext</td><td>AAGTTTCTCATCAACCATGC</td></tr>
<tr><td>hTRBV20-1_Int</td><td>ACTCTGACAGTGACCAGTGC</td></tr>
<tr><td>hTRBV23-1_Ext</td><td>GCGATTCTCATCTCAATGC</td></tr>
<tr><td>hTRBV23-1_Int</td><td>GCAATCCTGTCCTCAGAAC</td></tr>
<tr><td>hTRBV24-1_Ext</td><td>CCTACGGTTGATCTATTACTCC</td></tr>
<tr><td>hTRBV24-1_Int</td><td>GATGGATACAGTGTCTCTCGA</td></tr>
<tr><td>hTRBV25-1_Ext</td><td>ACTACACCTCATCCACTATTCC</td></tr>
<tr><td>hTRBV25-1_Int</td><td>CAGAGAAGGGAGATCTTTCC</td></tr>
<tr><td>hTRBV27_28_Ext</td><td>TGGTATCGACAAGACCCAG</td></tr>
<tr><td>hTRBV27_28_Int</td><td>TTC/|Y|/CCCTGAT/|Y|/CTGGAGTC</td></tr>
<tr><td>hTRBV29-1_Ext</td><td>TTCTGGTACCGTCAGCAAC</td></tr>
<tr><td>hTRBV29-1_Int</td><td>TCTGACTGTGAGCAACATGAG</td></tr>
<tr><td>hTRBV30_Ext</td><td>TCCAGCTGCTCTTCTACTCC</td></tr>
<tr><td>hTRBV30_Int</td><td>AGAATCTCTCAGCCTCCAGAC</td></tr>
</table>

TCR Constant α (TRAC) Reverse primer	
Internal(Int)/External (Ext)	Sequence
hTRAC_Ext	GACCAGCTTGACATCACAG
hTRAC_Int	TGTTGCTCTTGAAGTCCATAG

TCR Constant β (TRBC) Reverse primer	
Internal(Int)/External (Ext)	Sequence
hTRBC_Ext	TAGAACTGGACTTGACAGCG
hTRBC_Int	TTCTGATGGCTCAAACACAG

TCR Variable δ (TRDV) Forward primer	
Internal(Int)/External (Ext)	Sequence
HuTRDV1 Ext	GCCCAGAAGGTTACTCAAG
HuTRDV1 Int	AGCAAAGAGATGATTTTCCTTA
HuTRDV2 Ext	ATTGAGTTGGTGCCTGAAC
HuTRDV2 Int	TATATCAACTGGTACAGGAAGACC
HuTRDV3 Ext	TGTGACAAAGTAACCCAGAGTTC
HuTRDV3 Int	GGTACTGCTCTGCACTTACGAC
HuTRDV4/TRAV14Ext	CAAACCCAACCAGGAATG
HuTRDV4/TRAV14 Int	AGGAAAAGGAGGCTGTGAC
HuTRDV5/TRAV29 Ext	GCAAGTTAAGCAAAATTCACC
HuTRDV5/TRAV29 Int	CTGCTGAAGGTCCTACATTC
HuTRDV6/TRAV23 Ext	TTGATAGTCCAGAAAGGAGG
HuTRDV6/TRAV23 Int	CGTTTGACTACTTTCCATGG
HuTRDV7/TRAV36 Ext	GACAAGGTGGTACAAAGCC
HuTRDV7/TRAV36 Int	ATCTCTGGTTGTCCACGAG
HuTRDV8/TRAV38-2 Ext	CAGTCACTCAGTCTCAACCAG
HuTRDV8/TRAV38-2 Int	TCTGGTACAAGCAGCCTC

TCR Constant γ (TRDC) Reverse primer	
Internal(Int)/External (Ext)	Sequence
HuTRDC Ext	CTTCATATTTACCAAGCTTGACAG
HuTRDC Int	GATGACAATAGCAGGATCAAAC

TCR Variable γ (TRGV) Forward primer	
Internal(Int)/External (Ext)	Sequence
HuTRGV3.5 Ext	TCTTCCAACTTGGAAGGG
HuTRGV3.5 Int	GGTCATCTGCTGAAATCAC
HuTRGV7 Ext	TCTTCCAACTTGCAAGGG
HuTRGV7 Int	GGTCATCTGCTGTAATCACTTG
HuTRGVA Ext	GGGTCATCCTGTTTCCAG
HuTRGVA Int	TACCTAAGGACCTGTGTAGAGG
HuTRGVB Ext	TGGCCTCCCAAAGTACTG
HuTRGVB Int	TCCTCTTTCTATGTCCCAGG
HuTRGV8 Ext	CCAACTTGGAAGGGAGAAC
HuTRGV8 Int	AAAATGCCGTCTACACCC
HuTRGV9 Ext	CCAGGTCACCTAGAGCAAC
HuTRGV9 Int	TGTCCATTTCATATGACGG
HuTRGV10 Ext	TTATCAAAAGTGGAGCAGTTC
HuTRGV10 Int	CAGCTATCCATTTCCACGG
HuTRGV11 Ext	GAACAACCTGAAATATCTATTTCC
HuTRGV11 Int	CATATCTTGGAAGGCATCC
HuTRGV1.2.4.6 Ext	GGGTCATCTGCTGAAATCAC
HuTRGV1.2.4.6 Int	CCAGGAGGGGAAGGC

TCR Constant γ (TRGC) Reverse primer	
Internal(Int)/External (Ext)	Sequence
HuTRGC Int	CCCAGAATCGTGTTGCT
HuTRGC Ext	GGTGTTCCCCTCCTGG

Vector-Primer	
Forward (fwd), Reverse (rev	Sequence
pUC57_fwd	AGGCGATTAAGTTGGGTAAC
pUC57_rev	CGTATGTTGTGTGGAATTGTAG
pMSCV_fwd	TTGAACCTCCTCGTTCGACC
pMSCV_rev	CATATAGACAAACGCACACC
pET_Fwd	CTAGTTATTGCTCAGCGG
pET_Rev	CTAGTTATTGCTCAGCGG
