# Supplementary material for: A public human population of TRAV26+ type II natural killer T cells recognises CD1d in a lipid-independent manner
Source: Nat Commun. 2026 Oct 7;17:11430. doi: 10.1038/s41467-026-78124-5 (PMC13736892; doi:10.1038/s41467-026-78124-5)
Supplement: Supplementary file 2 [file 41467_2026_78124_MOESM2_ESM.pdf]

nature portfolio

Peer Review File

A public human population of TRAV26+ type II natural killer T cells recognises CD1d in a lipid-independent manner

Corresponding Author: Dr Catarina Almeida

This manuscript has been previously reviewed at another journal. This document only contains information relating to versions considered at Nature Communications.

This file contains all reviewer reports in order by version, followed by all author rebuttals in order by version.

Version 0:

Reviewer comments:

Reviewer #1

(Remarks to the Author)
This revised manuscript has addressed my comments and concerns. The expanded discussion of "Limitation of the study and future directions" strengthened the manuscript.

Reviewer #2

(Remarks to the Author)
I reviewed this manuscript previously for Nature Immunology and was enthusiastic about this then. I was happy with the excellent quality of the work and its originality. I raised a question about what the function of these type II NKT cells might be. The authors have responded by adding to the discussion some comments on monitoring the level of CD1d and also referring to intersting findings in Crohn's disease.
In my view they have responded well to the comments madeby the other reviewer and I remain enthusiastic about this manuscript.

Reviewer #3

(Remarks to the Author)
The authors have provided a thoughtful and thorough response to the critique, and as noted, the study is comprehensive. It is mildly disappointing that the authors suggest they could not find or chose not to search for, any primary cells with sufficient CD1d to activate the TRAV26+ T cells, although they suggest such cells exist. Regarding a different point, as the authors note, phenotypically and functionally characterizing the expanded cells could detect cell-expansion-specific artifacts. It is disappointing, however, that they did not stain the cells for PLZF. I believe this is the divider between two fundamental categories: innate-like T cells, such as iNKT cells and MAIT cells, and more adaptive type immune T cells. This is a simple experiment that would yield a big insight. PLZF is not activation-dependent.

Version 1:

Reviewer comments:

Reviewer #3

(Remarks to the Author)
The authors have made a very significant effort to address my concerns fully.

Open Access This Peer Review File is licensed under a Creative Commons Attribution 4.0 International License, which permits use, sharing, adaptation, distribution and reproduction in any medium or format, as long as you give appropriate credit to the original author(s) and the source, provide a link to the Creative Commons license, and indicate if changes were made.
In cases where reviewers are anonymous, credit should be given to 'Anonymous Referee' and the source.
The images or other third party material in this Peer Review File are included in the article's Creative Commons license, unless indicated otherwise in a credit line to the material. If material is not included in the article's Creative Commons license and your intended use is not permitted by statutory regulation or exceeds the permitted use, you will need to obtain permission directly from the copyright holder.
To view a copy of this license, visit https://creativecommons.org/licenses/by/4.0/

Response to reviewers' comments: manuscript NCOMMS-25-76080-T

Changes to original manuscript submission are highlighted in yellow

Reviewer 1

This revised manuscript has addressed my comments and concerns. The expanded discussion of "Limitation of the study and future directions" strengthened the manuscript.

No questions to address. We thank the reviewer for acknowledging our manuscript has been strengthened and suitable for publication.

Reviewer 2:

I reviewed this manuscript previously for Nature Immunology and was enthusiastic about this then. I was happy with the excellent quality of the work and its originality. I raised a question about what the function of these type II NKT cells might be. The authors have responded by adding to the discussion some comments on monitoring the level of CD1d and also referring to intersting findings in Crohn's disease.

In my view they have responded well to the comments madeby the other reviewer and I remain enthusiastic about this manuscript.

No questions to address. We thank the reviewer for acknowledging the high quality and originality of our improved manuscript, which adequately addressed all reviewer questions/concerns.

Reviewer 3:

The authors have provided a thoughtful and thorough response to the critique, and as noted, the study is comprehensive. It is mildly disappointing that the authors suggest they could not find or chose not to search for, any primary cells with sufficient CD1d to activate the TRAV26+ T cells, although they suggest such cells exist. Regarding a different point, as the authors note, phenotypically and functionally characterizing the expanded cells could detect cell-expansion-specific artifacts. It is disappointing, however, that they did not stain the cells for PLZF. I believe this is the divider between two fundamental categories: innate-like T cells, such as iNKT cells and MAIT cells, and more adaptive type immune T cells. This is a simple experiment that would yield a big insight. PLZF is not activation-dependent.

We thank the reviewer for recognising our "**thoughtful and thorough response to the critique"** of our comprehensive study.

Concern 1

It is mildly disappointing that the authors suggest they could not find or chose not to search for, any primary cells with sufficient CD1d to activate the TRAV26+ T cells, although they suggest such cells exist.

We acknowledge the opportunity to improve our manuscript and investigate whether circulating primary cells can lead to activation of TRAV26$^+$ cells.

B cells and monocytes are assumed to express higher levels of surface CD1d, when compared to T cells (Exley, immunology 2001). However, these are at much lower levels than those on C1R.CD1d cells (over 1-2 log difference as shown in our staining of healthy PBMCs performed on our previous resubmission to Nature Comms in 2025, alongside C1R.C1d, C1R.β2m$^{-/-}$ or C1R WT - Rebuttal Fig 2 then (shown below again as Rebuttal Figure 1).

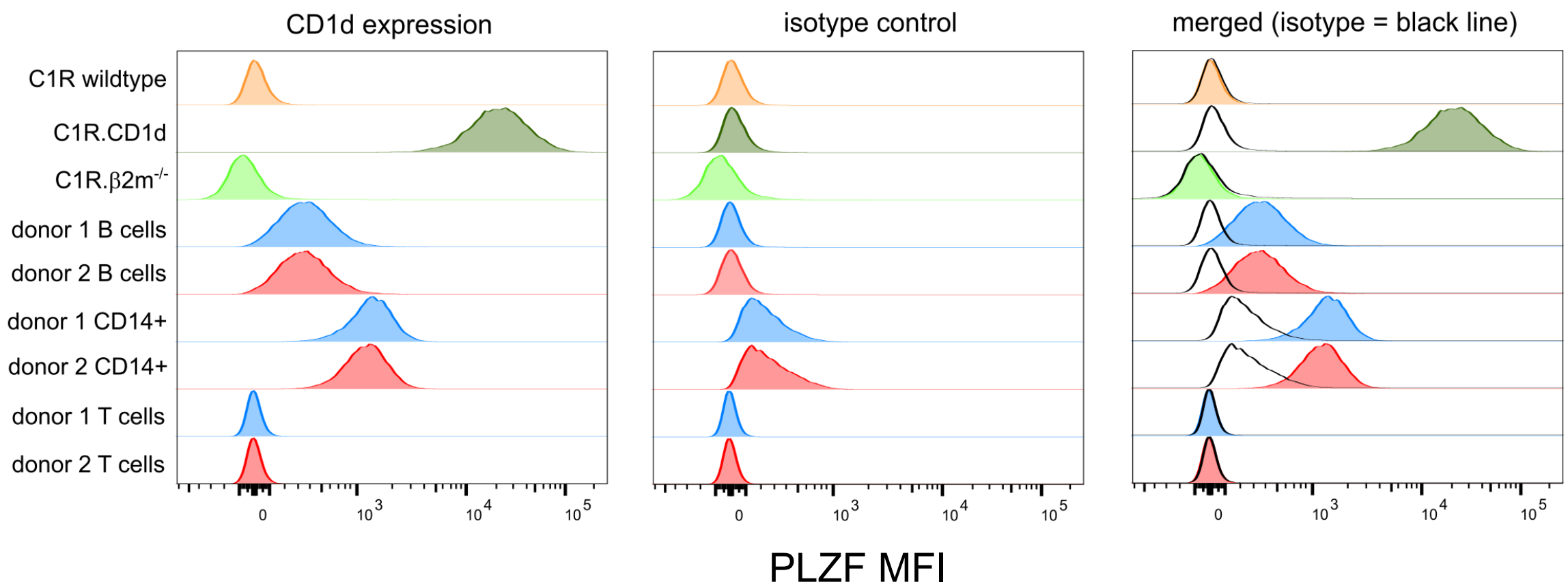

Rebuttal Fig. 1. CD1d expression on circulating B cells and CD14+ monocytes Flow cytometry offset overlay histograms comparing either the CD1d expression or level of background non-specific binding (isotype control) on human B cells, CD14+ monocytes and T cells from donor PBMC and C1R cells transduced to express high levels of CD1d (C1R.CD1d), CIR cells lacking CD1d (C1R.β2m-/-) and a parental C1R cells (C1R wildtype). Black lined histograms on the merged overlay are used to more directly evaluate true CD1d staining. Data is from 1 representative experiment of n=2 (each involving 2 donors), with only one including isotype controls (represented).

Given that CD1d-staining on CD14+ monocytes was the highest amongst these circulating cells, we have tested their ability to activate TRAV26+ NKT cells. To do this we co-cultured a TRAV26+ NKT reporter line (T26A) with primary CD14+ monocytes (magnetically enriched using anti-CD14 beads), or Monocyte derived dendritic cells (MoDCs), compared to co-cultures with C1R.CD1d, including a type I NKT line (NKT15) and α–GalCer or vehicle controls.

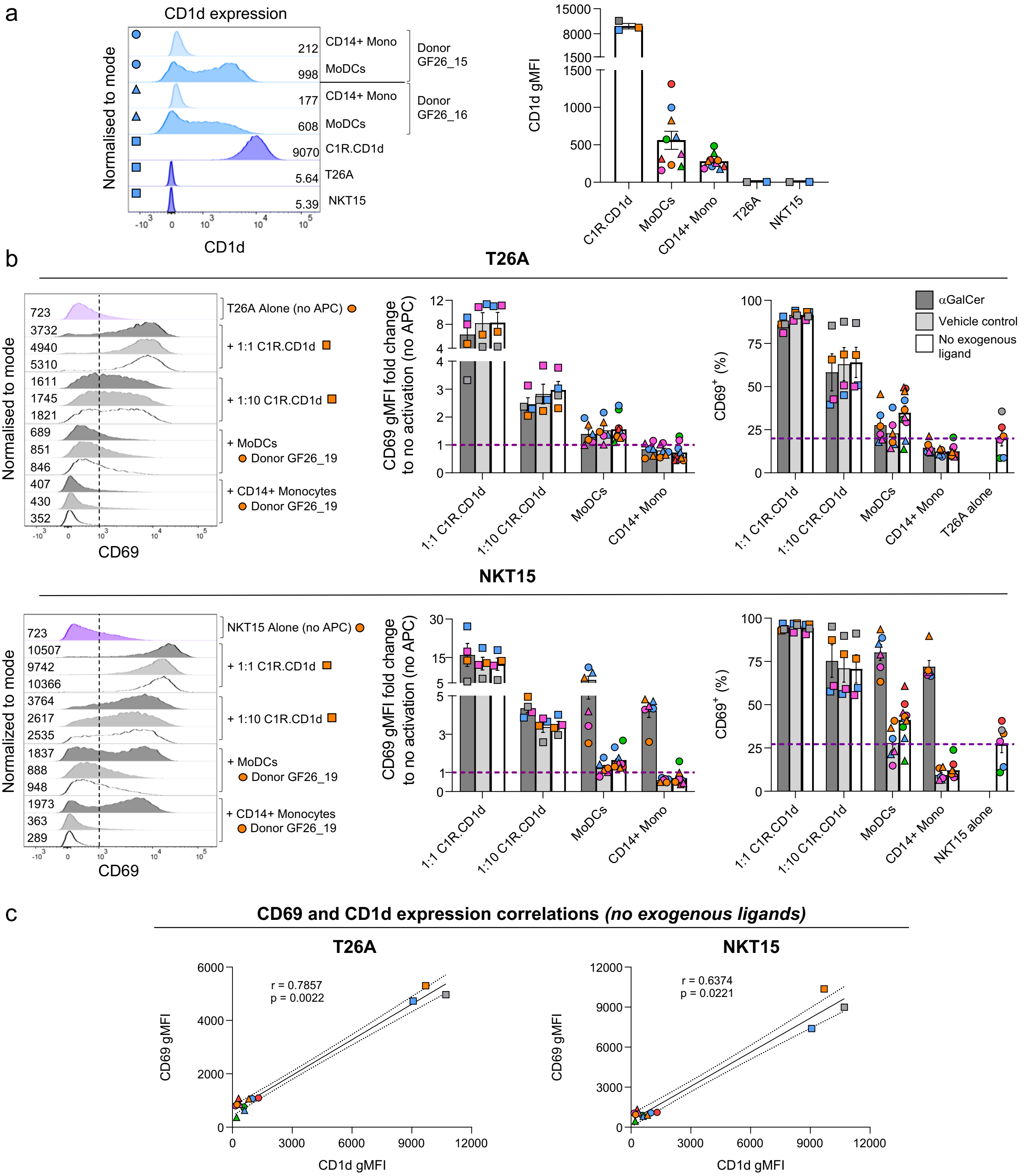

Rebuttal Figure 2 **(a)** Surface expression of CD1d was assessed on magnetically enriched CD14$^+$ monocytes, as well as monocyte-derived dendritic cells (MoDCs, day 5 post differentiation with GM-CSF/IL-4), CD1d-transduced C1R cells (C1R.CD1d), or T26A and NKT15 (SKW3.β2m$^{-/-}$). Histograms show CD1d geometric mean fluorescence intensity (gMFI, indicated by the numbers), from one representative experiment including two donor-derived CD14$^+$ Mono or MoDCs. Graphs show CD1d gMFI ± SEM from *n=5* experiments with CD14$^+$ Mono or MoDCs, each including two donors per experiment (represented by different shapes, 10 donors in total), *n=3* experiments for C1R.CD1d and *n=2* for each SKW3.β2m$^{-/-}$ NKT TCR reporter line. Conditions analysed alongside each experiment are matched by colour. **(b)** T26A or NKT15 were co-cultured with C1R.CD1d cells (at 1:1 or 1:10 antigen presenting cell (APC):effector ratios), and separately with donor-derived MoDCs or CD14$^+$monocytes (1:1 ratio), in the presence or absence of α-Galactosylceramide (α-GalCer; 0.33µg/mL), or vehicle control. Overlayed histograms show CD69 gMFI (indicated by numbers) from one representative

experiment. Vertical dashed line represents gate placement for CD69 % determination. Graphs show CD69 gMFI fold increase relative to each SKW3.$\beta 2m^{-/-}$ NKT reporter line alone (horizontal dashed purple line), or CD69 %, ± SEM. Data is from *n=4* experiments for all C1R:CD1d co-culture conditions, and *n=5* for $CD14^{+}$ Mono or MoDCs in the absence of antigen, *n=3* in the presence of α-GalCer or Vehicle. Data is shape and colour coded as per (**a**). **(c)** Spearman correlation analyses were performed between CD1d surface expression (CD1d gMFI) on APCs, including $CD14^{+}$ Mono, MoDCs or C1R.CD1d cells in 1:1 co-cultures in the absence of exogenous antigen, and the level of activation of each NKT TCR reporter lines (CD69 gMFI). Dotted lines represent simple linear regression with 95% confidence intervals. Data is derived from *n=3* experiments for C1R.CD1d and *n=5* for MoDCs (including 10 donors). Data is shape and colour coded as per (**a**).

Rebuttal Figure 2a (included in the main manuscript as Figure S4) shows that, following differentiation with GM-CSF and IL-4, $CD11c^{+}$ MoDCs showed a 2.1-4.4 fold increase in CD1d gMFI in 5 out of 10 donors, when compared to $CD14^{+}$ monocytes (pre-GM-CSF/IL-4 differentiation), suggesting that a subset of $CD1d^{+}$ MoDCs were expanded, or CD1d upregulated, perhaps reflecting different donor inflammatory status. Notably, CD1d levels were still lower than those CD1d-transfected C1Rs (8.3 to 69x lower gMFI).

In the absence of exogenous antigen, primary $CD14^{+}$ cells were unable to activate T26A cells when co-cultured at a 1:1 ratio, whereas MoDCs elicited a slight but consistent increase in CD69 gMFI by both T26A and NKT15, 1.1-2.4-fold, and 1.2-2.7 fold-increase, compared to no activation, respectively, reflecting 35% and 41% $CD69^{+}$ cells for each NKT line. However, this was inferior to the activation elicited by C1R.CD1d co-cultures co-cultured at similar 1:1 ratios, which led to 8.3-fold increases in CD69 gMFI mean for T26A (91% $CD69^{+}$) or 12.3 fold for NKT15 (94% $CD69^{+}$), or even when co-cultured at 1:10 C1R.CD1d:effector ratio (2.96-fold increase in CD69 gMFI / 64% $CD69^{+}$ for T26A, or 3.4 fold / 71% $CD69^{+}$ for NKT15). Panel C shows that indeed TCR-mediated activation of T26A (CD69 gMFI) significantly correlated to the levels of CD1d-surface expression on APCs, in the absence of exogenously added ligands.

In the presence of α-GalCer, both primary $CD14^{+}$ cells as well as *in vitro* differentiated MoDCs elicited activation of NKT15 (2.5-4.8 fold-increases in CD69 gMFI / 72% $CD69^{+}$, and 2.5-11.1 fold / 80% $CD69^{+}$, respectively), demonstrating that their CD1d surface levels are sufficient to allow α-GalCer presentation and TCR-dependent activation of type I NKT cells.

Altogether, our results suggest that CD1d-expression in primary circulating monocytes alone is likely insufficient to elicit TCR-dependent activation of CD1d-endo reactive cells on its own, although certain inflammatory settings driving higher CD1d-surface expression may be able to overcome this. This likely reflects an important physiological setting to prevent uncontrolled responses by CD1d-endo-reactive cells in circulation in healthy conditions.

A sentence about limitations of our study had already been added to the discussion in the previous resubmission, as well as a speculation on possible settings where CD1d overexpression has been reported, such as Hepatitis C infection, which may elicit activation of CD1d-endo reactive cells.

To accommodate for this new data in the main manuscript, (Figure S4), the gating strategies (Figure S1), discussion and methods have been adjusted.

Page 7 line 215 now reads:

We next assessed whether circulating $CD14^{+}$ monocytes, which constitutively express higher levels of CD1d, compared to T cells cells [36], could elicit activation of CD1d-endo reactive cells. To do this, we co-

cultured a TRAV26+ NKT reporter line (T26A) with primary CD14$^+$ monocytes (magnetically enriched using anti-CD14 beads), or donor matched CD11c$^+$ monocyte derived dendritic cells (MoDCs) and compared to co-cultures with C1R.CD1d, including a type I NKT line (NKT15) and α-GalCer or vehicle controls.

We detected clear CD1d surface expression (2.1-4.4 fold increase in CD1d gMFI) in MoDCs in 5 out of 10 donors, when compared to their pre-differentiation counterparts (CD14$^+$ Mono), suggesting that a subset of CD1d$^+$ MoDCs was being expanded, or CD1d upregulated, possibly reflecting different donor inflammatory status. Notably, CD1d levels were still lower than those CD1d-transduced C1Rs (8.3 to 69x lower gMFI).

In the absence of exogenous antigen, primary CD14$^+$ cells were unable to activate T26A cells when co-cultured at a 1:1 ratio, whereas MoDCs elicited a slight but consistent increase in CD69 gMFI by both T26A and NKT15, 1.1-2.4-fold, and 1.2-2.7 fold-increase, compared to no activation, respectively, reflecting 35% and 41% CD69$^+$ cells for each NKT line. However, this was inferior to the activation elicited by C1R.CD1d co-cultures co-cultured at similar 1:1 ratios, which led to 8.3-fold increases in CD69 gMFI mean for T26A (91% CD69$^+$) or 12.3 fold for NKT15 (94% CD69$^+$), or even when co-cultured at 1:10 C1R.CD1d:effector ratio (2.96-fold increase in CD69 gMFI / 64% CD69$^+$ for T26A, or 3.4 fold / 71% CD69$^+$ for NKT15). Panel C shows that indeed TCR-mediated activation of T26A (CD69 gMFI) significantly correlated to the levels of CD1d-surface expression on APCs, in the absence of exogenously added ligands.

In the presence of α-GalCer, both primary CD14$^+$ cells as well as *in vitro* differentiated MoDCs elicited activation of NKT15 (2.5-4.8 fold-increases in CD69 gMFI / 72% CD69$^+$, and 2.5-11.1 fold / 80% CD69$^+$, respectively), demonstrating that their CD1d surface levels are sufficient to allow α-GalCer presentation and TCR-dependent activation of type I NKT cells.

Altogether, our results suggest that CD1d-expression in primary circulating monocytes alone is likely insufficient to elicit TCR-dependent activation of CD1d-endo reactive cells on its own, although certain inflammatory settings driving higher CD1d-surface expression may be able to overcome this.

Methods Page 18 line 567 now reads:

Isolation of monocytes and generation of MoDCs for activation assays

CD14$^+$ were isolated from fresh PBMCs using magnetic enrichment in LS columns (Miltenyi) with anti-CD14 coated Microbeads (Miltenyi). To generate CD11c$^+$ MoDCs, enriched cells were plated at 2 x 10^6 cells per well in 24-well plates in 1.5 ml of in complete RPMI-1640 with GM-CSF (100 ng/ml) and IL-4 (50 ng/ml) (Biolegend). After 2 days, media was aspirated and replaced with fresh GM-CSF and IL-4 supplemented media. MoDCs were harvested at day 5 and assessed for CD1d and CD11c expression (anti-CD1d clone 51.1 Biolegend, anti CD11c clone B-ly6 BD). CD14$^+$ cells or CD11c$^+$ MoDCs were then co-cultured at a 1:1 ratio with T26A or NKT15, to test their ability to activate these cells, (CD69 upregulation after 16h), in the presence or absence of α-GalCer C26:0 at 0.33 μg/mL, or vehicle.

Discussion Page 14 line 434 now reads:

Thus, our results also suggest that some type II NKT cells might be poised to sense overall changes in surface expression of the Ag-presenting molecule CD1d during inflammatory or disease settings, rather than the lipid-Ag itself. This is illustrated by activation, albeit modest, of a TRAV26$^+$ line following increases in CD1d surface expression of *in vitro* differentiated MoDCs, compared to primary CD14$^+$ monocytes. Accordingly, changes in surface expression levels of CD1d have been shown to impact NKT

cell function [51] and are often associated with infection [52, 53, 54, 55], cancer [56] and autoimmunity [57]. For example, hepatocytes that are chronically infected with Hepatitis C display high CD1d surface expression relative to healthy tissue or other liver disease, to levels that resemble those of the C1R.CD1d transfectants that elicit IFN-γ release from intrahepatic CD1d-reactive T cells (type II NKT) [58]. Similarly, another study demonstrated that CD1d overexpression can be targeted by mouse CD1d-reactive Vγ4$^+$ T cells, promoting viral myocarditis *sequela* of coxsackievirus infections [59]. Whether these physiological contexts can lead to activation of TRAV26$^+$ NKT cells is an important question for further exploration.

Concern 2

Regarding a different point, as the authors note, phenotypically and functionally characterizing the expanded cells could detect cell-expansion-specific artifacts. It is disappointing, however, that they did not stain the cells for PLZF. I believe this is the divider between two fundamental categories: innate-like T cells, such as iNKT cells and MAIT cells, and more adaptive type immune T cells. This is a simple experiment that would yield a big insight. PLZF is not activation-dependent.

We acknowledge another opportunity to further improve our manuscript and investigate if the cells identified in our study share expression of the same master transcription regulator PLZF that defines the NKT and MAIT cell lineages.

We have now analysed PLZF expression of sorted and expanded CD1d-Endo tetramer$^+$ cells isolated from PBMCs from 6 healthy donors. After 21 days of expansion, PLZF expression was assessed upon re-gating on CD1d-Endo tetramer$^+$ from the (red), or CD1d-Endo tetramer- cells (grey). In all 6 donors screened, PLZF was consistently detected with statistical significance amongst CD1d-Endo-tetramer$^+$ cells (80-100%), with geometric mean fluorescence intensities that were 2-10-fold higher than those amongst CD1d-endo-tetramer$^-$ cells that had been sorted as non-tetramer binders and expanded in parallel (Rebuttal Figure 3). The later also contained some PLZF expressing populations (ranging 7-53%), perhaps reflecting the expansion of an unconventional T cell population, or a memory T cell subset. Overall, our results suggest that CD1d-Endo reactive cells, including those co-binding CD1d-α-GalCer tetramers (TRAV26$^+$) share functional and developmental similarities to type I NKT cells, and other innate-like T cell lineages such as MAIT cells and γδ T cells[1, 2].

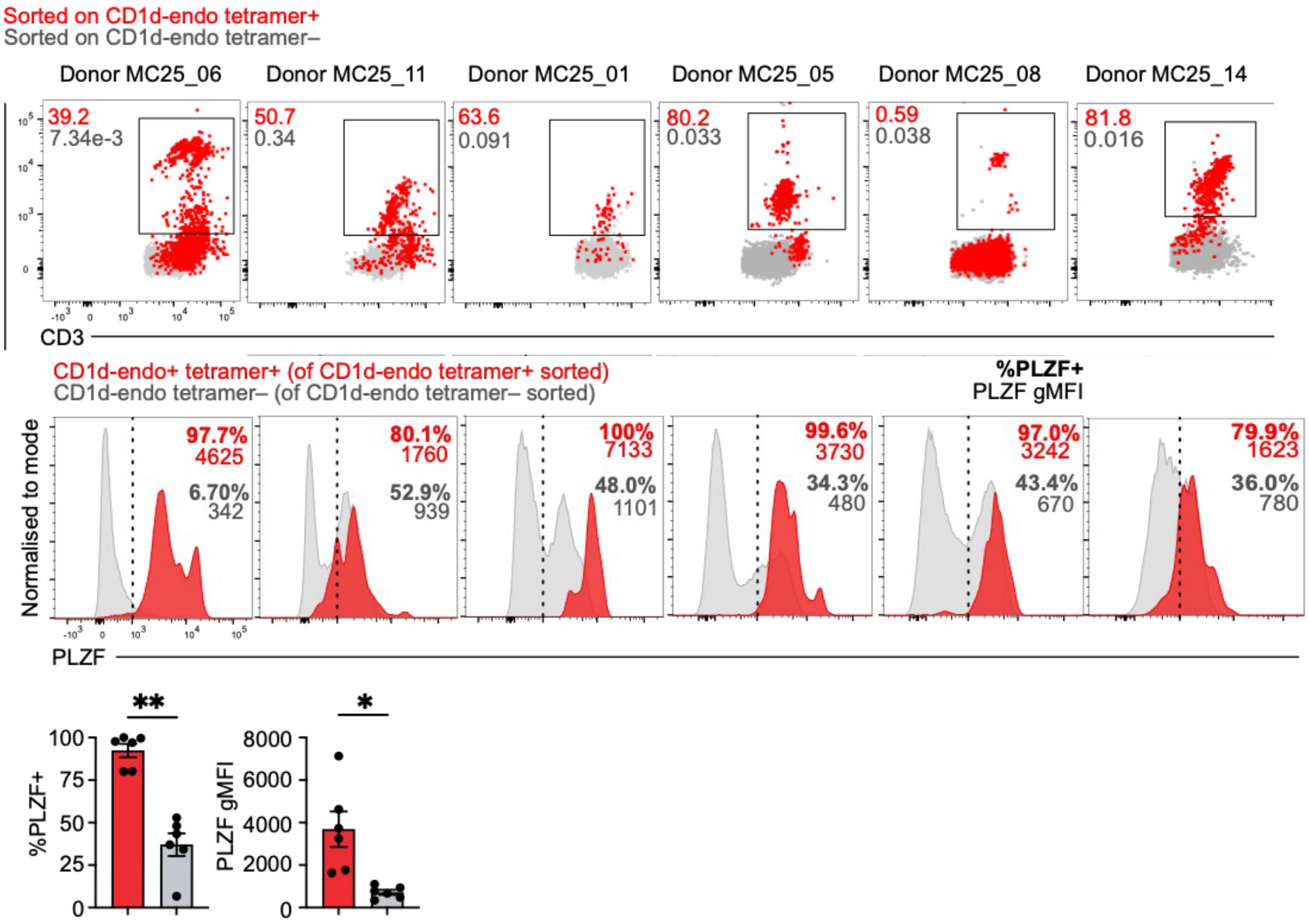

Rebuttal Figure 3 Overlay dot plots from 6 donors showing CD3 *versus* CD1d-endo tetramer (top) on cells that were expanded for 21 days after being sorted as CD1d-endo tetramer$^+$ CD3$^+$ (red) or CD1d-endo tetramer$^-$ CD3$^+$(grey). Numbers indicate percentages of cells within the CD1d-endo tetramer gates. Overlay histograms below show intracellular PLZF expression by cells gated as CD1d-endo tetramer$^+$ (in red, from CD1d-endo tetramer$^+$ sorted cells) or CD1d-endo tetramer$^-$ (in grey, from CD1d-endo tetramer$^-$ sorted cells). Numbers indicate PLZF % and gMFI below). Dashed line represents gate placement for % determination. Bar graphs summarise PLZF % and gMFI ± SEM from *n=6* donors analysed across 3 independent experiments. * P < 0.05, ** P < 0.01 for unpaired statistical t-test.

This data has been incorporated into the main manuscript (Figure 1F), and described within methods and discussion updated as follows:

Page 5 line 136 now reads

Next, we asked whether CD1d-Endo-tetramer sorted and expanded cells expressed promyelocytic leukemia zinc finger (PLZF), which is considered a master transcription factor associated with type I NKT cells and other innate-like T cell lineages[1, 2]. In all 6 donors screened, PLZF was consistently expressed by CD1d-Endo-tetramer$^+$ cells (80-100%), with geometric mean fluorescence intensities that were 2-10-fold higher than those of CD1d-endo-tetramer $^-$ cells that had been sorted as non-tetramer binders and expanded in parallel (Figure 1F). Notably, the latter also contained come PLZF$^+$ populations (ranging 7-53%), perhaps reflecting the expansion of an unconventional T cell population, or a memory T cell subset. Overall, our results suggest that CD1d-endo reactive cells, including those that co-bind CD1d-α-GalCer tetramer share functional and developmental similarities to type I NKT cells, and other innate-like T cell lineages such as MAIT cells and $\gamma\delta$T cells[1, 2].

Methods Page 17 line 517 now reads

Transcription factor intracellular staining was performed using Foxp3 / Transcription Factor Staining Buffer Set. (BD Biosciences) and anti-PLZF (R17-809 BD Horizon).

Discussion Page 15 line 469 now reads:

While this approach precludes addressing immune-function ex vivo, nonetheless, we have shown that expanded CD1d-endo tetramer$^+$ cells, including TRAV26 cells, can express PLZF and are capable of producing a range of cytokines, in particular TNF and IFNγ, implying that these cells share innate-like T cell features and have pro-inflammatory potential. Future studies will investigate their contributions to various health and disease states. It will also be important to address the intrinsic or extrinsic regulatory mechanisms that define the activity and immune function of TRAV26$^+$ type 2 NKT cells, including their reliance on secondary signals such as cytokines [65] or other receptors such as NK receptors [71], which can play a role in modulating activation status in both TCR-dependent and TCR-independent manners.
