# Supplementary material for: A public human population of TRAV26+ type II natural killer T cells recognises CD1d in a lipid-independent manner
Source: Nat Commun. 2026 Oct 7;17:11430. doi: 10.1038/s41467-026-78124-5 (PMC13736892; doi:10.1038/s41467-026-78124-5)
Supplement: Supplementary file 3 [file 41467_2026_78124_MOESM3_ESM.pdf]

nature portfolio

Corresponding author(s): Catarina F Almeida
jerome Le Nours
Daniel Pellicci

Last updated by author(s): 23/09/2026

Reporting Summary

Nature Portfolio wishes to improve the reproducibility of the work that we publish. This form provides structure for consistency and transparency in reporting. For further information on Nature Portfolio policies, see our Editorial Policies and the Editorial Policy Checklist.

Please do not complete any field with "not applicable" or n/a. Refer to the help text for what text to use if an item is not relevant to your study.
For final submission: please carefully check your responses for accuracy; you will not be able to make changes later.

Statistics

For all statistical analyses, confirm that the following items are present in the figure legend, table legend, main text, or Methods section.

n/a	Confirmed	
○	◉	The exact sample size (*n*) for each experimental group/condition, given as a discrete number and unit of measurement
○	◉	A statement on whether measurements were taken from distinct samples or whether the same sample was measured repeatedly
○	◉	The statistical test(s) used AND whether they are one- or two-sided<br>*Only common tests should be described solely by name; describe more complex techniques in the Methods section.*
◉	○	A description of all covariates tested
◉	○	A description of any assumptions or corrections, such as tests of normality and adjustment for multiple comparisons
○	◉	A full description of the statistical parameters including central tendency (e.g. means) or other basic estimates (e.g. regression coefficient) AND variation (e.g. standard deviation) or associated estimates of uncertainty (e.g. confidence intervals)
○	◉	For null hypothesis testing, the test statistic (e.g. *F*, *t*, *r*) with confidence intervals, effect sizes, degrees of freedom and *P* value noted<br>*Give P values as exact values whenever suitable.*
◉	○	For Bayesian analysis, information on the choice of priors and Markov chain Monte Carlo settings
◉	○	For hierarchical and complex designs, identification of the appropriate level for tests and full reporting of outcomes
◉	○	Estimates of effect sizes (e.g. Cohen's *d*, Pearson's *r*), indicating how they were calculated

Our web collection on statistics for biologists contains articles on many of the points above.

Software and code

Policy information about availability of computer code

Data collection	FacsDiva v9.0 (BD), Biacore T200 Control software 3.2 (Cytiva), Biacore 3000 Control software 3.1
Data analysis	FlowJo 10.8.1, GraphPad Prism Version 11, BIAevaluation Version 3.1 (Biacore AB), Biacore T200 Evaluation Software 3.2.1 (Biacore AB), XDS,

For manuscripts utilizing custom algorithms or software that are central to the research but not yet described in published literature, software must be made available to editors and reviewers. We strongly encourage code deposition in a community repository (e.g. GitHub). See the Nature Portfolio guidelines for submitting code & software for further information.

Data

Policy information about availability of data

All manuscripts must include a data availability statement. This statement should provide the following information, where applicable:
- Accession codes, unique identifiers, or web links for publicly available datasets
- A description of any restrictions on data availability
- For clinical datasets or third party data, please ensure that the statement adheres to our policy

The coordinates of the T26A NKT TCR-CD1d-SM and T26A NKT TCR-CD1d-GD3 ternary complexes were deposited in the Protein Data Bank (PDB) database under the accession codes 8SGB and 8SGM, respectively.

Research involving human participants, their data, or biological material

Policy information about studies with human participants or human data. See also policy information about sex, gender (identity/presentation), and sexual orientation and race, ethnicity and racism.

Reporting on sex and gender	Neither sex or gender were used to stratify donor samples. De-identified buffy coats from healthy individuals provided by the
Reporting on race, ethnicity, or other socially relevant	Neither sex or gender were used to stratify donor samples. De-identified buffy coats from healthy individuals provided by the Australian Red cross
Population characteristics	Australian healthy blood donors, Age, diagnostic, treatment or genotypic information of donor samples were not collected.
Recruitment	Donor PBMC samples were recruited ad-hoc via Australian Red Cross Blood Service (agreement 13-04VIC-07, 22-11VIC-04 and
Ethics oversight	Buffy coat samples were obtained from the Australian Red Cross after approval from the University of Melbourne Human

Note that full information on the approval of the study protocol must also be provided in the manuscript.

Field-specific reporting

Please select the one below that is the best fit for your research. If you are not sure, read the appropriate sections before making your selection.

- [x] Life sciences
- [ ] Behavioural & social sciences
- [ ] Ecological, evolutionary & environmental sciences

Life sciences study design

All studies must disclose on these points even when the disclosure is negative.

Sample size	Stated in each Figure legend. Fig 1A: 33 donors post-Tame, 37 post sort enrichment. Sample sizes and number of independent experiments were
Data exclusions	No data was excluded from the analysis of experiments shown within the manuscript and associated files.
Replication	Experimental repeats stated in each figure legend. All experiments were included in the final analysis, and all replications successful.
Randomization	All donor samples were paired (i.e. samples were treated and untreated as controls) therefore random assignment to groups was not performed.
Blinding	Blinding was not applied and was not relevant for this study. The processes involved knowing and keeping track of cell lines/reagents/samples.

Behavioural & social sciences study design

All studies must disclose on these points even when the disclosure is negative.

Study description	
Research sample	
Sampling strategy	
Data collection	
Timing	
Data exclusions	
Non-participation	
Randomization	

Ecological, evolutionary & environmental sciences study design

All studies must disclose on these points even when the disclosure is negative.

Study description	
Research sample	
Sampling strategy	
Data collection	
Timing and spatial scale	
Data exclusions	
Reproducibility	
Randomization	

Blinding

Did the study involve field work? ◯ Yes ◯ No

Field work, collection and transport

Field conditions

Location

Access & import/export

Disturbance

Reporting for specific materials, systems and methods

We require information from authors about some types of materials, experimental systems and methods used in many studies. Here, indicate whether each material, system or method listed is relevant to your study. If you are not sure if a list item applies to your research, read the appropriate section before selecting a response.

Materials & experimental systems

n/a	Involved in the study
	◉ Antibodies
	◉ Eukaryotic cell lines
◉	Palaeontology and archaeology
◉	Animals and other organisms
◉	Clinical data
◉	Dual use research of concern
◉	Plants

Methods

n/a	Involved in the study
◉	ChIP-seq
	◉ Flow cytometry
◉	MRI-based neuroimaging

Antibodies

Antibodies used: CD3ε (UCHT1, eBioscience and Becton Dickinson), CD4 (RPA-T4, BD Pharmingen), CD8α (SK1, BD Pharmingen), CD19 (HIB19, BioLegend),

Validation: All antibodies are commercially sourced, validated, and titrated in-house before use in these experiments - dilutions as per

Eukaryotic cell lines

Policy information about cell lines and Sex and Gender in Research

Cell line source(s): Expi293 cells were purchased from ThermoFisher Scientific. C1R, SKW3, HEK293T cells (GNTI) HEK-293S were historically

Authentication: Cell lines were not authenticated, other then sequence verification of gene inserts (TCRs/CD1d) or KO (b2m).

Mycoplasma contamination: Cell lines, including HEK293T, SKW3.b2mko, C1Rs were negative at time of testing

Commonly misidentified lines (See ICLAC register): No misidentified lines were used in this study

Palaeontology and Archaeology

Specimen provenance

Specimen deposition

Dating methods

☐ Tick this box to confirm that the raw and calibrated dates are available in the paper or in Supplementary Information.

Ethics oversight

Note that full information on the approval of the study protocol must also be provided in the manuscript.

Animals and other research organisms

Policy information about studies involving animals; ARRIVE guidelines recommended for reporting animal research, and Sex and Gender in Research

Laboratory animals

Wild animals

Reporting on sex

Field-collected samples

Ethics oversight

Note that full information on the approval of the study protocol must also be provided in the manuscript.

Clinical data

Policy information about clinical studies

All manuscripts should comply with the ICMJE guidelines for publication of clinical research and a completed CONSORT checklist must be included with all submissions.

Clinical trial registration

Study protocol

Data collection

Outcomes

Dual use research of concern

Policy information about dual use research of concern

Hazards

Could the accidental, deliberate or reckless misuse of agents or technologies generated in the work, or the application of information presented in the manuscript, pose a threat to:

No	Yes	
☒	☐	Public health
☒	☐	National security
☒	☐	Crops and/or livestock
☒	☐	Ecosystems
☒	☐	Any other significant area

Experiments of concern

Does the work involve any of these experiments of concern:

No	Yes	
☒	☐	Demonstrate how to render a vaccine ineffective
☒	☐	Confer resistance to therapeutically useful antibiotics or antiviral agents
☒	☐	Enhance the virulence of a pathogen or render a nonpathogen virulent
☒	☐	Increase transmissibility of a pathogen
☒	☐	Alter the host range of a pathogen
☒	☐	Enable evasion of diagnostic/detection modalities
☒	☐	Enable the weaponization of a biological agent or toxin
☒	☐	Any other potentially harmful combination of experiments and agents

Plants

Seed stocks

Novel plant genotypes

Authentication

ChIP-seq

Data deposition

- [ ] Confirm that both raw and final processed data have been deposited in a public database such as GEO.
- [ ] Confirm that you have deposited or provided access to graph files (e.g. BED files) for the called peaks.

Data access links
May remain private before publication.

Files in database submission

Genome browser session
(e.g. UCSC)

Methodology

Replicates

Sequencing depth

Antibodies

Peak calling parameters

Data quality

Software

Flow Cytometry

Plots

Confirm that:

- [x] The axis labels state the marker and fluorochrome used (e.g. CD4-FITC).
- [x] The axis scales are clearly visible. Include numbers along axes only for bottom left plot of group (a 'group' is an analysis of identical markers).
- [ ] All plots are contour plots with outliers or pseudocolor plots.
- [x] A numerical value for number of cells or percentage (with statistics) is provided.

Methodology

Sample preparation	As described in the methods. PBMCs from healthy donor buffy coats were prepared through density gradient centrifugation
Instrument	BD LSRFortessa II™ with or BD FACSAria III™ (BD)
Software	FACSDiva v9.0 (BD) was used for data collection. FlowJo v10.8.1 (BD) was used for analysis
Cell population abundance	When reassessed immediately after the first-sort, purities ranged 13-65 %. Stable TCR transduced lines were selected for having
Gating strategy	Live SKW3.b2m cells or HEK293T cells were identified by FSC-A vs SSC-A. Doublets were removed by FSC-A vs FSC-H

- [x] Tick this box to confirm that a figure exemplifying the gating strategy is provided in the Supplementary Information.

Magnetic resonance imaging

Experimental design

Design type

Design specifications

Behavioral performance measures

Acquisition

Imaging type(s)

Field strength

Sequence & imaging parameters

Area of acquisition

Diffusion MRI ◯ Used ◯ Not used

Preprocessing

Preprocessing software

Normalization

Normalization template

Noise and artifact removal

Volume censoring

Statistical modeling & inference

Model type and settings

Effect(s) tested

Specify type of analysis: ◯ Whole brain ◯ ROI-based ◯ Both

Statistic type for inference

(See Eklund et al. 2016)

Correction

Models & analysis

n/a	Involved in the study
◯	◯ Functional and/or effective connectivity
◯	◯ Graph analysis
◯	◯ Multivariate modeling or predictive analysis

Functional and/or effective connectivity

Graph analysis

Multivariate modeling and predictive analysis

This checklist template is licensed under a Creative Commons Attribution 4.0 International License, which permits use, sharing, adaptation, distribution and reproduction in any medium or format, as long as you give appropriate credit to the original author(s) and the source, provide a link to the Creative Commons license, and indicate if changes were made. The images or other third party material in this article are included in the article's Creative Commons license, unless indicated otherwise in a credit line to the material. If material is not included in the article's Creative Commons license and your intended use is not permitted by statutory regulation or exceeds the permitted use, you will need to obtain permission directly from the copyright holder. To view a copy of this license, visit http://creativecommons.org/licenses/by/4.0/
